# Biosynthesis of pathogen-induced hydroxylated tryptamine derivatives in barley

**DOI:** 10.1093/plphys/kiag622

**Published:** 2026-08-22

**Authors:** Joachim Møller Christensen, Mette Marie Toldam-Andersen, Hans Jørgen Lyngs Jørgensen, Nikola Micic, Nanna Bjarnholt, Mohammed Saddik Motawia, Mallika Vijayanathan, Mathilde Pizaine, Jan Günther, Louise Kjaerulff, Dan Staerk, Meike Burow, Sara Thodberg, Elizabeth H J Neilson

**Affiliations:** Department of Plant and Environmental Sciences, The University of Copenhagen, Thorvaldsensvej 40, 1871 Frederiksberg C, Denmark; Department of Plant and Environmental Sciences, The University of Copenhagen, Thorvaldsensvej 40, 1871 Frederiksberg C, Denmark; Department of Plant and Environmental Sciences, The University of Copenhagen, Thorvaldsensvej 40, 1871 Frederiksberg C, Denmark; Department of Plant and Environmental Sciences, The University of Copenhagen, Thorvaldsensvej 40, 1871 Frederiksberg C, Denmark; Department of Plant and Environmental Sciences, The University of Copenhagen, Thorvaldsensvej 40, 1871 Frederiksberg C, Denmark; Department of Plant and Environmental Sciences, The University of Copenhagen, Thorvaldsensvej 40, 1871 Frederiksberg C, Denmark; Department of Plant and Environmental Sciences, The University of Copenhagen, Thorvaldsensvej 40, 1871 Frederiksberg C, Denmark; Department of Plant and Environmental Sciences, The University of Copenhagen, Thorvaldsensvej 40, 1871 Frederiksberg C, Denmark; National College of Chemical Engineering, Université de Haute-Alsace, 3 rue Alfred Werner, Mulhouse 68200, France; Department of Plant and Environmental Sciences, The University of Copenhagen, Thorvaldsensvej 40, 1871 Frederiksberg C, Denmark; Department of Drug Design and Pharmacology, The University of Copenhagen, Jagtvej 160, 2100 København Ø, Denmark; Department of Drug Design and Pharmacology, The University of Copenhagen, Jagtvej 160, 2100 København Ø, Denmark; Department of Plant and Environmental Sciences, The University of Copenhagen, Thorvaldsensvej 40, 1871 Frederiksberg C, Denmark; Department of Plant and Environmental Sciences, The University of Copenhagen, Thorvaldsensvej 40, 1871 Frederiksberg C, Denmark; Department of Plant and Environmental Sciences, The University of Copenhagen, Thorvaldsensvej 40, 1871 Frederiksberg C, Denmark

## Abstract

Barley (*Hordeum vulgare*), a major cereal crop, suffers significant yield losses due to pathogen attack. Understanding the chemical defense responses that follow pathogen infection is essential to uncover new resistance targets in barley. In this study, we employed an untargeted metabolomics and mass spectrometry imaging approach to profile the chemical landscape of barley leaves inoculated with the hemibiotrophic fungal pathogen *Pyrenophora teres* f. *teres*, the causal agent of net blotch. Pathogen infection triggered a significant chemical defense response, including the induction of many phenylpropanoids and alkaloids. Specifically, a strong induction of tryptophan-derived metabolites was observed, including tryptamine, serotonin, and the novel 2-oxo-tryptamine, which accumulated at infection sites. Integration of transcriptomic-driven pathway discovery with biochemical characterization identified a flavin-containing monooxygenase and a cytochrome P450 (CYP71P10) involved in 2-oxo-tryptamine and serotonin biosynthesis, respectively. Our results show increased metabolic investment toward indolic compounds in barley leaves following infection, and activation of these pathways across different pathogens and cultivars suggests a conserved resistance mechanism. This study provides new insights into the barley defense response, offering new metabolic targets for the development of disease-resistant cereal crops.

## Introduction

Barley (*Hordeum vulgare* L.) is the fourth most cultivated cereal crop, for food, fodder, and raw materials. An estimated average of 15% of barley yield is lost per annum due to pathogens (Oerke and Dehne 2004), but this loss can be up to 40% depending on the pathogen and environmental conditions (Clare et al 2020). Global warming alters the range of phytopathogens to which plants are exposed, as well as their susceptibility to these pathogens. Consequently, climate change leads to an overall increase in disease pressure (Sangiorgio et al 2020; Seth and Sebastian 2024). Coupled with the drastically increasing world population, estimated to reach 9 billion by 2050, and the associated rise in food demand, there is an urgent need to expand our knowledge on plant–pathogen interactions at both molecular and systemic levels (Raza et al 2019).

Plants produce a large array of bioactive specialized defense metabolites that provide chemical protection against pathogen attack. Plant defense metabolites are broadly categorized into phytoalexins and phytoanticipins (VanEtten et al 1994). Phytoalexins are synthesized de novo in response to biotic challenges such as pathogen attack or herbivore infestation, while phytoanticipins are constitutively present in plant tissues, acting as preemptive protective agents. Barley produces many different specialized metabolite classes, including benzofurans, hydroxynitrile glucosides (HNGs), terpenoids, phenylamides, and alkaloids, each with varying modes of action. For example, benzofurans (ie hordatines) possess antifungal activity and accumulate in the seedling leaves (Hamany Djande et al 2022), providing early-stage protection (Stoessl and Unwin 1970), but can also be induced in mature plants (Laupheimer et al 2023). Similarly, levels of barley-specific diterpenes (ie hordedanes) are induced after infection and exuded from barley roots, exhibiting antifungal activity and modifying fungal root colonization (Liu et al 2024). Tryptophan-derived metabolites represent a further metabolite class, highly relevant for defense in barley and other important cereal crops. Specifically, the tryptophan-derived metabolites tryptamine, serotonin, and phenylamides (eg triticamides) consistently accumulate in barley after infection by a wide range of pathogenic hosts (Ube et al 2019a, 2019b; Lemcke et al 2021). Barley also produces the tryptophan-derived indole-alkaloid gramine, 5,5′-dihydroxy-2,4′-bitryptamine (a dehydrodimer of serotonin), and 3-(2-aminoethyl)-3-hydroxyindolin-2-one (AEHI; di-hydroxylated tryptamine derivative), both of which have reported bioactivity (Corcuera 1984; Wippich and Wink 1985; Ishihara et al 2017; Lu et al 2018).

Despite significant progress toward identifying and measuring the chemical defense constituents produced by barley in response to pest and pathogen attack, biosynthetic pathway elucidation within barley is limited to a few compound classes: hydroxynitrile glucosides (Knoch et al 2016), hordedane diterpenes (Liu et al 2024), and the indole alkaloid gramine (Leland and Hanson 1985; Dias et al 2024; Ishikawa et al 2024). Transcriptomic studies show significant upregulation of genes leading to tryptamine biosynthesis, but downstream assessment of where this metabolic flux leads remains unknown (Lemcke et al 2021). In contrast to other members of the Poaceae family, cultivated barley (ie *H. vulgare*) does not produce the indole-derived defense class of benzoxazinoids (BXs) (Ube et al 2017). In other grasses, the core BX pathway initiates with the formation of indole by the action of an indole-3-glycerol phosphate lyase (BX1), a diversified homolog of the alpha subunit of tryptophan synthase (Zhuang et al 2012). Indole then undergoes 4 consecutive hydroxylations, leading to the first considered benzoxazinoid (ie DIBOA) by 4 different CYP71C enzymes (BX2–5), co-occurring in a tightly regulated gene cluster (Frey et al 1997; Wu et al 2022). In a case of convergent evolution, the first 2 hydroxylations (ie BX2 and BX3) are catalyzed by class B flavin-containing monooxygenases (FMOs) in the BX-producing eudicots *Consolida orientalis*, *Lamium galeobdolon*, and *Aphelandra squarrosa* (Florean et al 2023; Florean et al 2025). Although barley lacks BXs, it still possesses orthologous *BX2–4* genes (Sue et al 2011), as well as several related FMOs, that are significantly induced after infection (Sjokvist et al 2019). In the absence of a *BX1* gene (and BXs), it is speculated that barley has evolved a defense pathway that is channeled toward a tryptophan-derived metabolic defense response in contrast to indole-derived metabolites observed for other cereals.

In this study, we subject the elite malting barley cultivar RGT Planet to *Pyrenophora teres f. teres* (henceforth *P. teres*) infection (the causal agent of net form net blotch) to characterize its metabolic response. *P. teres* is a major hemibiotrophic fungal pathogen that can result in up to 40% yield loss in barley (Clare et al 2020). Untargeted metabolomic analysis identified tryptamine, serotonin, and 2 novel hydroxylated tryptamine derivatives to be significantly induced after *P. teres* infection, with the most abundant hydroxylated tryptamine isomer identified as 2-oxo-tryptamine (2OT). A combination of metabolomics, transcriptomics, and heterologous expression in *Nicotiana benthamiana* and *Escherichia coli* identified an FMO from the N-OX3 family as an enzyme that catalyzes the production of 2OT from tryptamine, and we hereby name it 2-oxo-tryptamine synthase (2OTS). Furthermore, we biochemically characterize CYP71P10, a tryptamine 5-hydroxylase, as the serotonin-producing gene in barley. These results suggest that the rapid induction of tryptamine-derived metabolites constitutes an important metabolic branch of the barley defense response to pathogen attack.

## Results

### Disease progression of net blotch in barley

**Figure 1:**
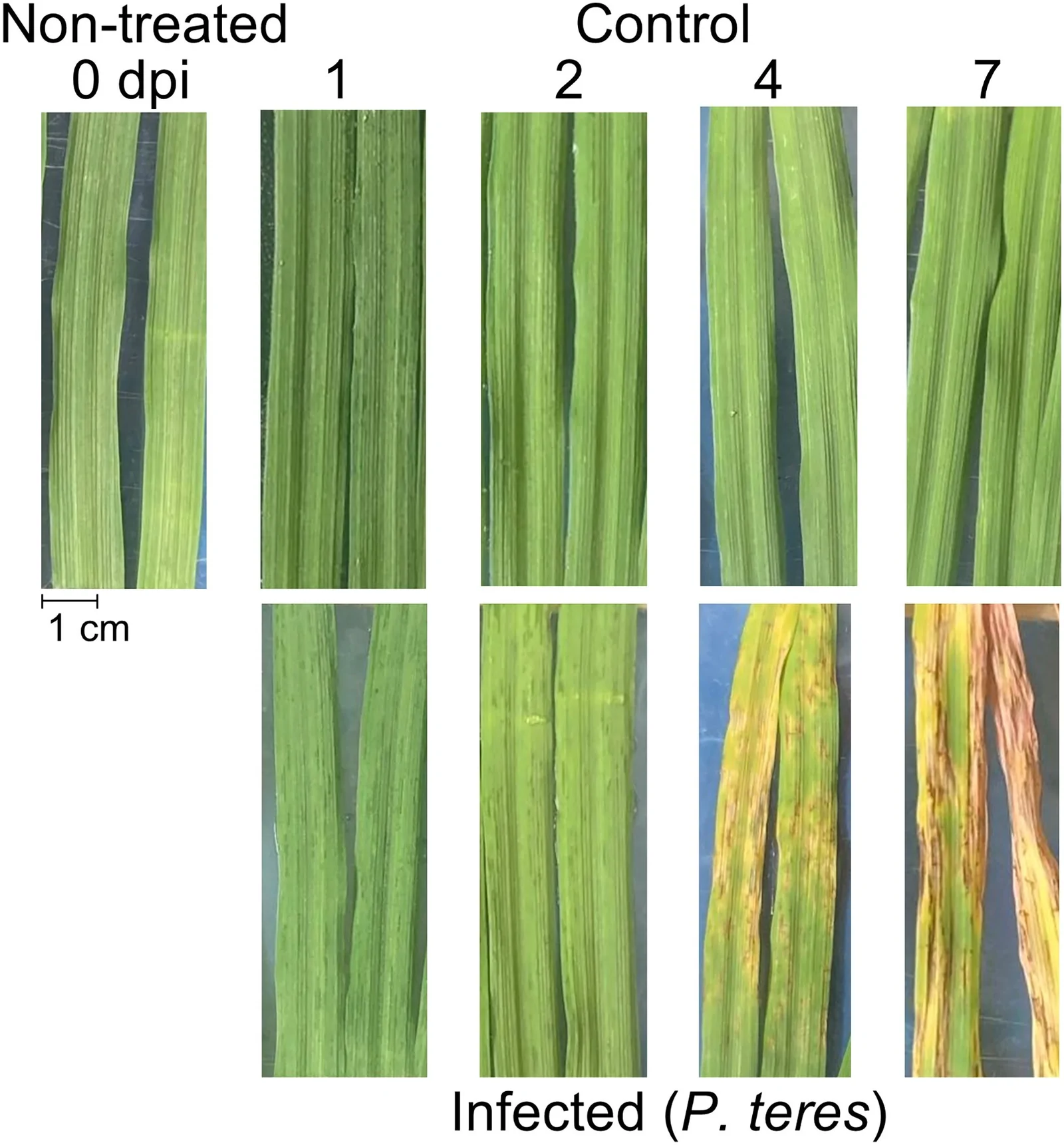
Disease progression of net blotch on barley cv. RGT Planet leaves inoculated with *P. teres*. Development of disease symptoms at 0 (non-treated), 1, 2, 4, and 7 dpi. Representative pictures of infected and control leaves.

In this study, barley cv. RGT Planet was infected with the hemibiotrophic fungal pathogen *P. teres*, the causal agent of net blotch. Progression of disease symptoms was observed visually for non-treated, control, and infected leaves at 0, 1, 2, 4, and 7 days post inoculation (dpi). Clear signs of infection were noticeable (Fig. 1). At 2 dpi, symptoms of fungal infection were already apparent, with infected leaves appearing more yellow than controls and exhibiting longitudinal necrotic lesions characteristic of the disease (Tini et al 2022). At 4 dpi, infected leaves showed brown necrotic lesions surrounded by chlorotic areas, with chlorotic areas increasing in size, leading to wilting and leaf death at 7 dpi. No disease symptoms were detected on the control leaves.

### Chemical profiling of barley leaves following infection by the fungus *P. teres*

An untargeted metabolomics approach was used to investigate the chemical profile of barley leaves in response to *P. teres* infection. Leaf methanol extracts were analyzed by ultra-high-performance liquid chromatography coupled with quadrupole time-of-flight tandem mass spectrometry (UHPLC-qTOF-MS/MS) and subsequently processed in MZmine4. The generated feature table and spectral files were used as input for various metabolomic computational tools, including feature-based molecular networking (FBMN), SIRIUS, and CANOPUS (Fig. S1). Prior to MZmine4 data processing, the overall reliability of the spectral data acquisition throughout the analysis was assessed and confirmed by principal component analysis, which showed narrow clustering of quality control (QC) pooled samples and blanks (Fig. S2).

**Figure 2:**
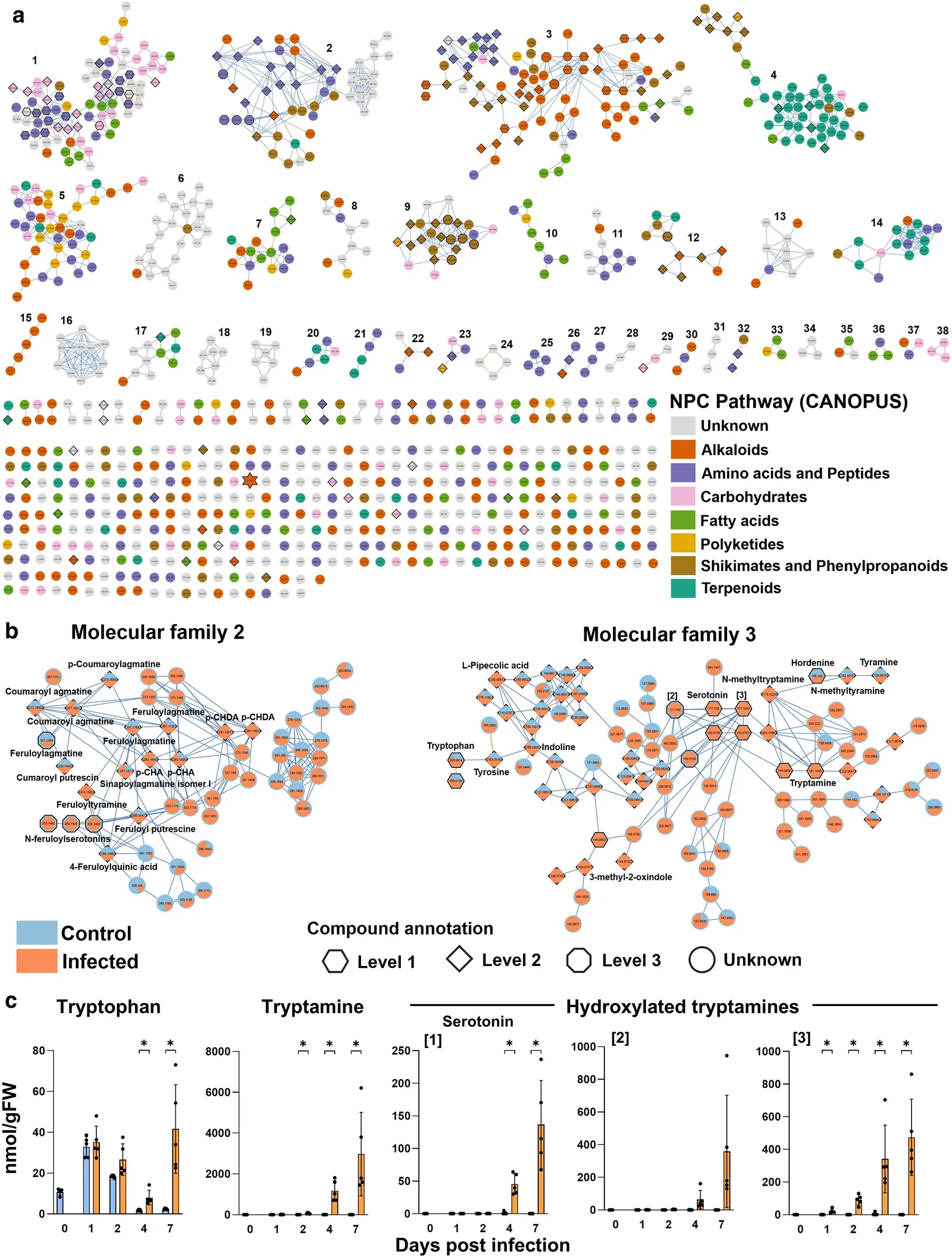
Chemical profiling of barley leaves after fungal infection by *P. teres.* a) Global FBMN visualizing the detected chemical space of barley cv. RGT Planet leaves under infected and control conditions at 0, 1, 2, 4, and 7 dpi constructed from all detected features across all samples (*n* = 5). The 38 largest molecular families (ie ≥ 3 nodes) in the network are numbered. Nodes are colored according to predicted chemical compound classes (NPC pathway categories) assigned by CANOPUS. The shape corresponds to the level of compound annotation. The star represents AEHI (level 1). b) Enlarged depiction of molecular families 2 and 3 highlighting features (primarily phenylpropanoids and alkaloids) that are highly induced following *P. teres* infection. Pie charts indicate relative abundance of individual features in control (blue) and infected (orange) samples across the entire spectral dataset. c) Abundance (nmol g^−1^ fresh weight) of tryptophan, tryptamine, and 3 hydroxylated tryptamine derivatives, which are strongly induced after pathogen inoculation. Data are presented as mean ± SD (*n* = 5). All compounds were quantified using authentic standard curves; the unknown hydroxytryptamine [2] was quantified using an authentic serotonin standard. Asterisks (*) indicate *P* < 0.05 from unpaired Student's *t* tests comparing control and infected samples at each time point for each metabolite. AEHI, 3-(2-aminoethyl)-3-hydroxy-indolin-2-one (Ishihara et al. 2017); NPC, NPClassifier taxonomy (Kim et al. 2021); *p*-CHA, *p*-coumaroyl-hydroxyagmatine; *p-*CHDA, *p*-coumaroyl-hydroxydehydroagmatine.

FBMN compares and distinguishes between chromatographic features, which are then connected to one another based on spectral similarity. Each detected feature is depicted as a node in the network, and related nodes are grouped in molecular families (MFs) (Nothias et al 2020). The FBMN generated from the entire barley spectral dataset (non-treated, control, and infected leaves) comprised 998 nodes, each representing detected metabolite features with MS2 spectral information. In the network, 631 nodes clustered into 75 MFs (2 or more connected nodes) based on their spectral similarity (Fig. 2a). The remaining 367 nodes were represented as singletons (ie not included in an MF).

Seventy-six features were removed from the generated feature table during blank subtraction, resulting in a total of 922 features. Semiautomated putative annotation of 106 metabolites was achieved with confidence level 2 (Sumner et al 2007; Schymanski et al 2014) by matching (>0.7 cosine score) the processed liquid chromatography–tandem MS (LC-MS/MS) data output to the publicly available spectral libraries Global Natural Product Social Molecular Networking (GNPS) and MassBank of North America (MoNA). This annotated metabolite list corresponds to an annotation rate of approximately 12% of the detected features. The metabolite annotation rate was further improved to 14% by matching 25 features to in-house available authentic standards (confidence level 1) analyzed with the same LC-MS/MS method as the samples. Additionally, annotated features in the molecular network could, in some cases, be used as anchoring nodes to propagate the information and manually annotate structurally similar metabolites in the same MF by comparison to spectral data reported in the literature (confidence level 2). For example, *p*-coumaroyl-hydroxyagmatine and *p*-coumaroyl-hydroxydehydroagmatine (*p*-CHDA) are found in MF 2 (von Röpenack et al 1998), along with a methoxychalcone in MF 12 (Ube et al 2021). Lastly, annotations of the network were propagated by the addition of in silico structure predictions by SIRIUS (confidence level 3). These different approaches resulted in a 16.5% annotation rate of the detected features (Table S1). The annotated features represented 12 different MFs in the network; thus, for 26 MFs, no features were putatively annotated. Additionally, in some cases (eg MFs 16, 18, and 19), it was not even possible for CANOPUS to predict the compound class based on the MS/MS spectra. Collectively, this demonstrates that the barley metabolome is highly complex and largely unknown.

CANOPUS was applied for de novo chemical compound class predictions using the NPClassifier taxonomy (Kim et al 2021). Overall, our results demonstrate that barley synthesizes a broad range of specialized metabolite classes, including a diverse array of terpenoids, phenylpropanoids, shikimates, and alkaloids (Fig. 2a). Terpenoids (MF 4) were largely unaffected by *P. teres* infection, with the exception of a putatively identified glucosylated conjugate of β-ionone (feature ID 3321; *m/z* 373.222 [M + H]^+^), which was significantly induced at 7 dpi (*P* = 0.001). In contrast, the production of many different phenylpropanoids, alkaloids, and shikimates (ie MFs 2 and 3) was induced during pathogen infection (Fig. 2b).

Known barley metabolites were observed in the current study, with varying responses detected after infection. For example, all 5 HNGs (epiheterodendrin, epidermin, sutherlandin, osmaronin, and dihydroosmaronin; MF 1), as well as the phenethylamine alkaloids hordenine and N-methyltyramine (MF 3), were detected but did not significantly change in concentration in response to pathogen infection (Fig. S3). In contrast, L-pipecolic acid (MF 3), *N*-hydroxy pipecolic acid glucoside (MF 26), and several methoxychalcones (MF 12) were detected only in the *P. teres*–treated barley leaves. In MF 12, 3 methoxychalcone and flavanone features were annotated by spectral library matching as 3,4,2′,4′,6′-pentamethoxychalcone, naringenin 5,7-dimethyl ether, and 2′,4-dihydroxy-3,4′,6′-trimethoxychalcone. Based on these annotations, we were able to further identify 2 neighboring nodes as 2′-hydroxy-3,4,4′,6′-tetramethoxychalcone and 4,6,2″,4″-tetramethoxychalcone 2″-beta-glucoside at confidence levels 2 and 3 by comparison to spectral information reported by Ube et al (2021) and in silico structure prediction by SIRIUS, respectively (Table S1).

### Production of tryptophan-derived specialized metabolites is strongly induced in barley after *P. teres* infection

Significant alteration in metabolism from 4 dpi as a response to infection by *P. teres* was observed, as well as ontogenetic variation (Fig. S4). Assessment of LC-MS base peak chromatograms identified several peaks significantly induced after infection (Fig. S5). In terms of absolute abundance, several tryptophan-derived specialized metabolites — particularly, tryptamine (*m/z* 161.10 [M + H]^+^ and *m/z* 144.08 [M-NH_3_ + H]^+^), and 3 apparent hydroxylated tryptamine derivatives (*m/z* 177.10 [M + H]^+^ and *m/z* 160.08 [M-NH_3_ + H]^+^) — were among the highest induced metabolites detected in our experimental setup. Mean ± SE tryptamine levels increased from being below the level of detection at 0 dpi to 2,970 ± 915 nmol/g fresh weight (FW) at 7 dpi, while the 3 different hydroxy tryptamine isomers were strongly induced from 0.28 ± 0.02, 0.34 ± 0.03, and nondetectable at 0 dpi to a mean ± SE of 137 ± 30, 359 ± 154, and 475 ± 104 nmol/g FW during *P. teres* infection, respectively (Fig. 2c, Fig. S5).

**Figure 3:**
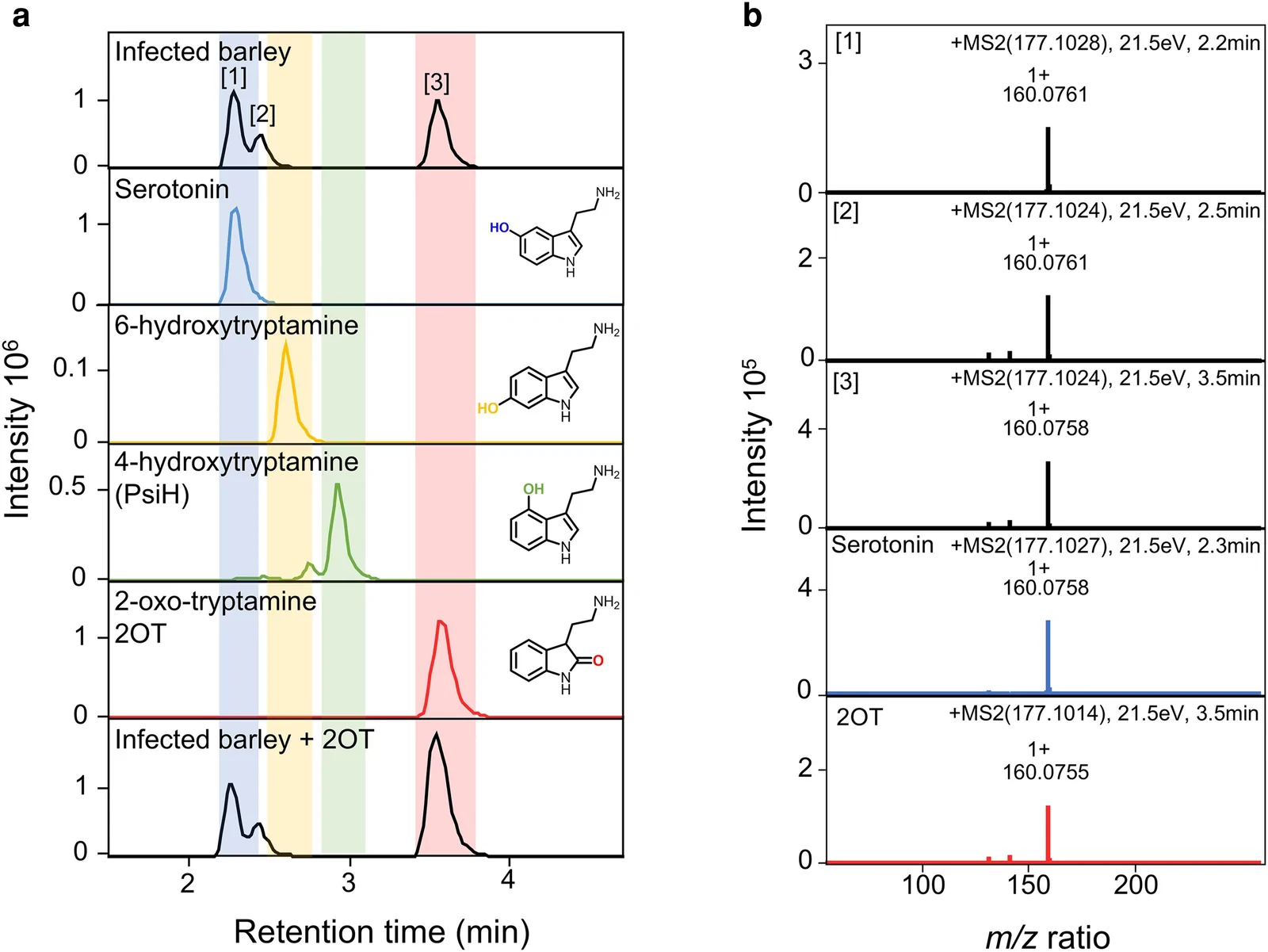
Production of serotonin and 2-oxo-tryptamine in barley leaves after fungal infection is confirmed by authentic standards. a) Extracted ion chromatograms (EICs) (177.10; 160.08) matching the dominant ions of hydroxylated tryptamine. Top panel is a methanol extract of barley leaves (cv. RGT Planet) infected with *P. teres* 7 dpi. Panels 2, 3, and 5 are authentic standards of positional isoforms of hydroxylated tryptamine derivatives: serotonin (5-hydroxytryptamine; blue), 6-hydroxytryptamine (yellow), and 2OT (red), the spontaneous tautomer of 2-hydroxytryptamine. Panel 4 is a methanol extract from *N. benthamiana* leaves expressing the 4-hydroxytryptamine (green) producing cytochrome P450 (PsiH), fed with tryptamine 4 days post infiltration. Panel 6 is the extract from panel 1 spiked with 2OT. b) MS2 fragmentation pattern for the parent ion (*m/z* 177.10 [M + H]^+^) of hydroxylated tryptamine isomers for peaks [1], [2], and [3] and the authentic standards of serotonin (blue) and 2OT (red).

To determine the identity of the hydroxylated tryptamine derivatives, the acquired LC-MS data were mined in more detail. The 3 features all exhibited near-identical fragmentation patterns but eluted at distinct retention times (Fig. 3), leading to the hypothesis of different positional isomers of hydroxytryptamine. Authentic standards for serotonin, 6-hydroxytryptamine, *N*-hydroxytryptamine, 3-(2-aminoethyl)-1H-indol-1-ol, and 2OT were obtained and/or chemically synthesized, whereby the chemical synthesis of 3-(2-aminoethyl)-1H-indol-1-ol is reported for the first time. Furthermore, 4-hydroxytryptamine was obtained by transiently expressing PsiH, a characterized cytochrome P450 (Fricke et al 2017) involved in biosynthesizing psilocybin in the fungus *Psilocybe cubensis*, in *N. benthamiana*, and co-feeding it with tryptamine. Peak 1 from the infected sample coeluted with authentic serotonin (Fig. 3), supporting previous findings that serotonin production is induced after pathogen infection in barley (Ishihara et al 2017). Authentic 2OT coeluted with peak 3 in the infected barley leaves (Fig. 3). To further confirm the structure of this novel metabolite, peak 3 was purified and analyzed by nuclear magnetic resonance (NMR) spectroscopy (Fig. S6) and represents the first report of this metabolite from a biological system. The tested standards did not coelute at the retention time of the remaining serotonin isomer (ie peak [2]), and in the experimental setup used, molecular ions of the 2 *N*-hydroxylated tryptamine derivatives [*N*-hydroxytryptamine and 3-(2-aminoethyl)-1H-indol-1-ol] were not detected with our LC-MS method and likely degraded. Authentic standards for the remaining α, β, 3′, and 7′ tryptamine hydroxylation positions were either unavailable or restricted.

### Localization of hydroxylated tryptamine derivatives and other induced metabolites by matrix-assisted laser desorption/ionization mass spectrometry imaging

**Figure 4:**
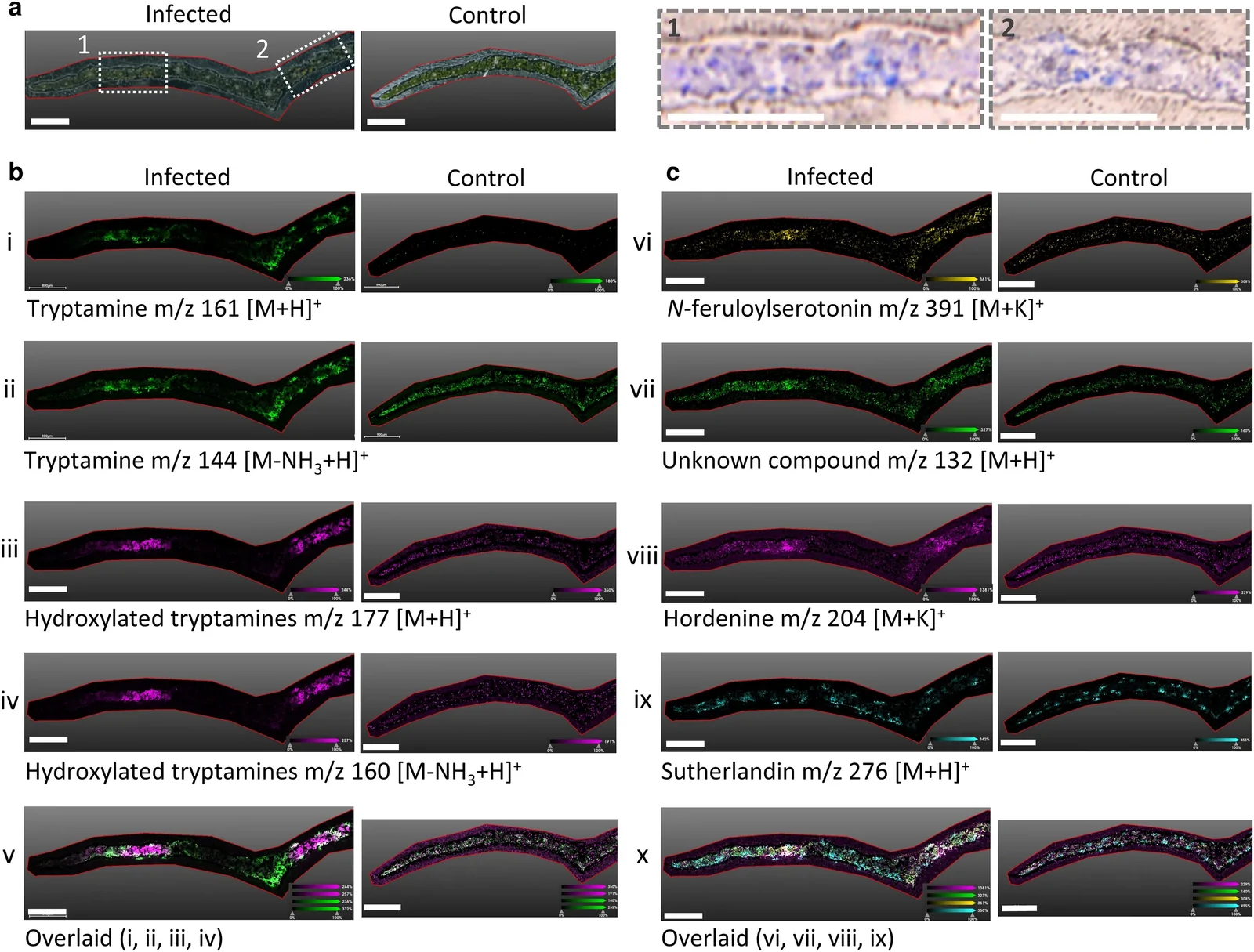
MALDI-MSI localization of selected metabolites in barley leaf cross sections after *P. teres* infection at 4 dpi with a spatial resolution of 20 µm. a) Scanned images of representative infected and control barley leaves (cv. RGT Planet) at 4 dpi, with yellow necrotic tissue at the site of infection highlighted in the 2 dotted boxes. On the right, corresponding regions are color inverted with increased magnification, and necrotic tissue is indicated in blue. b) Localization of tryptamine and hydroxylated tryptamine derivatives. Tryptamine (green) signals correspond to (i) *m/z* 161.1073 [M + H]^+^ and (ii) *m/z* 144.0808 [M-NH_3_ + H]^+^ ions. Hydroxylated tryptamine derivatives (magenta) are detected as isobaric signals corresponding to (iii) *m/z* 177.1025 [M + H]^+^ and (iv) 160.0757 [M-NH_3_ + H]^+^ ions. Overlay (v) of tryptamine and hydroxylated tryptamine derivatives shows localization in the middle and outer regions of infected tissue, respectively. c) Selected barley phytoalexins and phytoanticipins. The phytoalexins (vi) *N-*feruloylserotonin (yellow; *m/z* 391.1049 [M + K]^+^) and (vii) an unknown compound (green; compound ID 2191, *m/z* 132.0450 [M + H]^+^) are localized to the infected tissue regions. Signals corresponding to phytoanticipins (viii) hordenine (pink; m/z 204.0785 [M + K]) and (ix) sutherlandin (cyan; *m/z* 276.1077 [M + H]^+^) are detected in both control and infected leaf samples. Hordenine and sutherlandin are mainly detected in mesophyll and epidermal regions, respectively, although hordenine has a higher ion intensity at the sites of infection, as shown in the overlay image (x). Scale bar corresponds to 800 µm and 900 µm in infected and control samples, respectively. Color bars show the intensity of detected ions. All images are generated with a ±5 mDa error interval.

To investigate where 2OT and serotonin accumulate, matrix-assisted laser desorption/ionization mass spectrometry imaging (MALDI-MSI) was performed on control and *P. teres*–infected leaves, sampled at 4 dpi (*n* = 3). Ions corresponding to tryptamine (*m/z* 161.1073 [M + H]^+^; 144.0808 [M-NH_3_ + H]^+^), 2OT, and serotonin (*m/z* 177.1025 [M + H]^+^; 160.0757 [M-NH_3_ + H]^+^) were detected and confirmed by independently assessing authentic standards in co-crystallization analysis. To differentiate between serotonin and 2OT, tims (trapped ion mobility separation) analysis was performed on authentic standards and the plant tissue. Both serotonin and 2OT ions [M-NH_3_ + H]^+^ measured a shared tims mobility value of 0.6 (Fig. S7a), so they could not be used for ion differentiation on the plant tissue. The protonated 2OTS ion [M + H]^+^ did measure a unique mobility value of 0.6, but no clear differentiation between the serotonin and 2OT ions could be determined due to a low signal intensity observed for 2OT using this method (Fig. S7b). In control samples, tryptamine localized to mesophyll tissue, while trace levels of the ions corresponding to 2OT and serotonin were detected in the epidermal layer. After infection, ions corresponding to tryptamine and hydroxylated tryptamine derivatives were colocalized at the site of infection and found in the mesophyll tissue regions showing necrotic lesions and discoloration (Fig. 4, Fig. S8). However, within the infected tissue, tryptamine and hydroxylated tryptamine derivatives showed different localization in the middle and outer regions, respectively.

To identify and investigate the localization of other key metabolites induced after *P. teres* infection, random forest analysis was performed to rank the most important metabolite variables based on their contribution to model accuracy at 4 dpi (Fig. S9, Table S2). Within the top 15 most important contributing metabolites, 3 annotated metabolites were identified corresponding to 3,4,2′,4′,6′-pentamethoxychalcone, *N*-feruloylserotonin, and tryptamine (MFs 12, 2, and 3, respectively). MALDI-MSI analysis showed that 2 of these metabolites, *N-*feruloylserotonin (*m/z* 391.1049 [M + K]^+^) and an unknown compound (feature ID 2191; *m/z* 132.0450 [M + H]^+^), were also produced at the site of infection (Fig. S10). In contrast, the phytoanticipins and apparently uninduced metabolites hordenine and the hydroxynitrile glucoside sutherlandin showed an even distribution in the mesophyll and clustered epidermal cells, respectively (Fig. 4). Interestingly, an increased ion intensity of hordenine was detected at the infection sites. As overall hordenine concentrations did not differ between control and infected leaves (*P* = 0.57), this localization pattern may suggest metabolite relocation. Three other hydroxynitrile glucosides (dihydroosmaronin, epiheterodendrin, and epidermin; *m/z* 300.0844 [M + K]^+^) shared the same localization pattern as sutherlandin. Osmaronin (*m/z* 298.0687 [M + K]^+^), however, showed a different localization pattern where the corresponding signal of its potassium adduct was also present in the mesophyll tissue of control and infected leaves (Fig. S11). MALDI-MSI analysis also indicated a fine-tuned temporal and spatial response, as a region of tissue around the main midrib was found to accumulate tryptamine and the 2 phytoalexins prior to obvious necrotic lesions, and without colocalization of the biochemically downstream hydroxylated tryptamine derivatives. The other imaged biological replicates did not show accumulation of tryptamine and other metabolites at the midrib (or within vascular bundles; Figs. S8, S10, and S12), so this accumulation does not suggest a localization associated with transport.

### Characterization of 2OTS and CYP71P10 (T5H)

**Figure 5:**
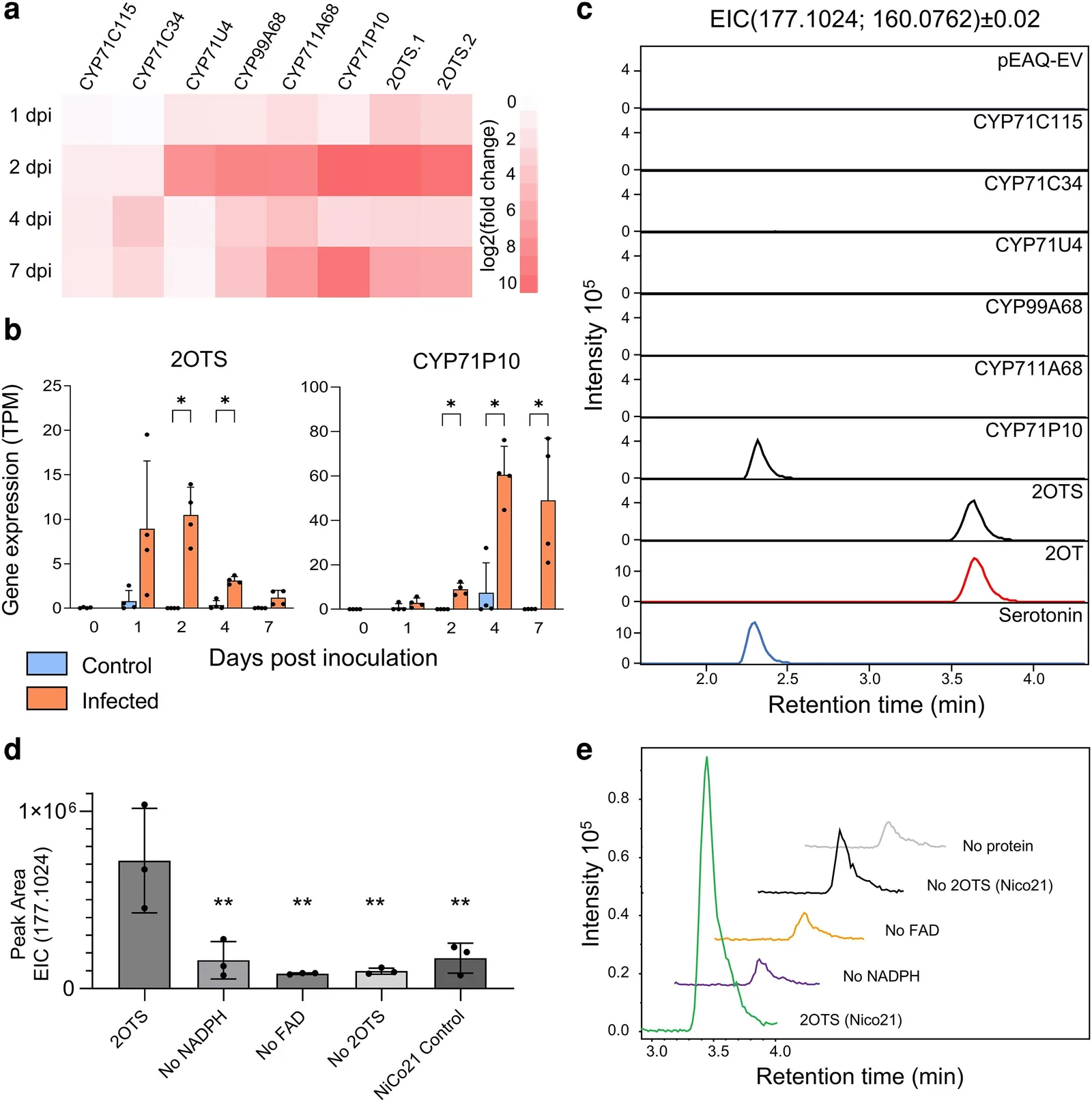
Characterization of hydroxylated tryptamine derivatives in *N. benthamiana* and *E. coli.* a) Gene expression heatmap of log2-transformed fold change between infected barley (cv. RGT Planet) and control at 4 different time points: 1, 2, 4, and 7 dpi. b) Transcript per million (TPM) value gene expression profile of 2 selected gene candidates, CYP71P10 (T5H) and 2OTS, at 0, 1, 2, 4, and 7 dpi in both control and infected samples (*n* = 4); asterisks denote significance by unpaired *t* test at *P* < 0.05. c) Extracted ion chromatograms (EICs) for the dominant ions of hydroxylated tryptamine-derived metabolites *m/z* 177.1024 [M + H]^+^ and *m/z* 160.0762 [M-NH_3_ + H]^+^ from methanolic extracts of *N. benthamiana* plants expressing 7 different monooxygenase candidates fed with tryptamine 4 d post infiltration and authentic standards for 2OT (red) and serotonin (blue). d) Peak area of ions *m/z* 177.1024 [M + H]^+^ and *m/z* 160.0762 [M-NH_3_ + H]^+^, corresponding to 2OT levels (± SD), measured in an in vitro activity assay of recombinant 2OTS supplied with tryptamine, with or without cofactors FAD and NADPH (*n* = 3). Background activity was assessed using a crude lysate from the untransformed NiCo21 cells. Double asterisks (**) indicate adjusted *P* < 0.01 from Dunnett's multiple comparisons test following ordinary 1-way ANOVA, comparing each non–full protein assay to the full protein assay. e) Representative EICs of the dominant ions of 2OT (*m/z* 177.1024 [M + H]^+^ and *m/z* 160.0762 [M-NH_3_ + H]^+^) from the recombinant enzymatic assay.

To identify potential tryptamine oxidases, a stepwise approach was used. First, transcriptome analysis was performed on RNA extracted from barley leaves infected with *P. teres* sampled at 0, 1, 2, 4, and 7 dpi. Given that a significant increase in 2OT and tryptamine was established at 4 and 7 dpi, respectively (Fig. 2c), candidate monooxygenases were selected based on a log2 fold change cutoff of 2, late in the infection (ie 4 and 7 dpi). Specifically, CYPs and FMOs were targeted, previously shown to hydroxylate the indole backbone (Frey et al 1997; Florean et al 2025). This analysis resulted in 76 CYPs and 6 FMOs identified as potential candidates. To narrow the candidate list, a coexpression network using known genes for tryptamine biosynthesis was generated using PlantNexus (Zhou et al 2022; Fig. S13a, b) and assessed for the presence of the identified coexpressed monooxygenase enzymes using a mutual rank cutoff of 50. This resulted in the identification of 3 CYPs (CYP711A69, HORVU.RGT_PLANET.PROJ.4HG00366880, HORVU4Hr1G079620; CYP71U4, HORVU.RGT_PLANET.PROJ.7HG00602210, HORVU7Hr1G043540; and CYP99A68, HORVU.RGT_PLANET.PROJ.2HG00090280, HORVU2Hr1G004610) and a class B FMO from the N-OX3 family (HORVU.RGT_PLANET.PROJ.7HG00671660; HORVU7Hr1G121260) (Fig. 5a; Jiang et al 2025). The identified FMO was expressed as 2 isoforms, differing by only 2 amino acids (Met and Gly) at the start codon, resulting in a slightly longer N-terminus. Accordingly, the isoforms were treated as the same gene for further characterization. An identified CYP71P10 exhibited a log2 fold change of approximately 10 at 2 dpi, and expression followed serotonin accumulation (Figs. 2c and 5a, b). A corresponding barley HORVU gene identifier for this CYP71P10 does not exist in the first annotation of the barley cv. Morex genome, and therefore coexpression could not be assessed using PlantNexus (this tool uses MorexV1 annotations). However, as the identified barley CYP71P10 (HORVU.RGT_PLANET.PROJ.6HG00496350) is orthologous to the previously characterized tryptamine 5-hydroxylase CYP71P1 from rice (Fujiwara et al 2010), this gene was included to assess its ability to hydroxylate tryptamine to form serotonin. Finally, candidate selection was expanded to include an orthologous gene approach, with 2 selected pathogen-induced members of the CYP71C family included for further characterization: CYP71C115 (HORVU.RGT_PLANET.PROJ.6HG00503910; HORVU6Hr1G024850) and CYP71C34 (HORVU.RGT_PLANET.PROJ.3HG00205600; HORVU3Hr1G011870), despite having a coexpression mutual rank >50. The CYP71C family has been shown to hydroxylate indole for benzoxazinoid biosynthesis in other grasses (Frey et al 1997; Frey et al 2009).

To test the activity of the 7 candidate genes, *Agrobacterium tumefaciens*–mediated transient expression was performed in *N. benthamiana* leaves. Leaves were supplied with exogenous tryptamine 4 d after infiltration, followed by a methanolic extraction of metabolites 5 d after infiltration. LC-MS analysis of assay extracts showed that all heterologously expressed monooxygenases produced metabolite signals distinct from the empty-vector control (Fig. S14), indicating general catalytic activity. However, hydroxylation of tryptamine was observed exclusively for 2 enzyme candidates, CYP71P10 and 1 FMO (Fig. 5c).

Extracts from *N. benthamiana* plants expressing the CYP71P10 contained a feature matching the *m/z* and retention time of serotonin (Fig. 5c), and we therefore conclude that the barley CYP71P10 is a tryptamine-5-hydroxylase (T5H). The metabolic extracts from plants expressing the FMO (HORVU.RGT_PLANET.PROJ.7HG00671660; HORVU7Hr1G121260) contained a feature matching the *m/z*, retention time, and fragmentation pattern of 2OT (Fig. 5c). Heterologous expression of this FMO in *E. coli*, combined with in vitro crude enzymatic assays, demonstrated increased production of 2OT compared to controls, corroborating the in vivo experiment (Fig. 5d, e). We therefore denote the identified FMO as 2OTS.

2OTS possesses canonical class B FMO features: 2 paired Rossmann folds (G*X*G*XX*G) (CATH 3.50.50.60) binding FAD and NADPH and the FMO identifying motif (FXGXXXHXXXY/F) (Thodberg and Jakobsen Neilson 2020). A BLAST search against the members of the phylogenetic tree of the FMO N-OX family (Jiang et al 2025) revealed 2OTS to be part of the N-OX3 family. An analysis of 2OTS in cv. RGT Planet showed that it possesses 7 gene copies (>98% identity) that are positioned in a tandem repeat gene cluster at the far end of chromosome 7 (Table S3). To assess whether any natural *Hordeum* accessions lack this FMO, we identified 2OTS gene orthologs across the pan-genome containing 76 accessions, including cultivated, landrace, and wild *Hordeum* members (Jayakodi et al 2024). A total of 341 genes with >98% amino acid sequence identity to 2OTS were considered gene copies (Table S4). All accessions within the pan-genome possess multiple copies of 2OTS, with gene copy numbers ranging from 2 to 8, all positioned at the end of pseudochromosome 7 in their respective genome assemblies (Table S3).

Given that several of the tested monooxygenase genes are coexpressed and induced in barley leaves (Figs. S13b, 5a), it was hypothesized that they may work together in concert to produce additional, novel metabolites. However, no additional products were observed when 2OTS, CYP71C115, CYP71C34, CYP71U4, CYP711A68, and CYP99A68 were transiently expressed together, with and without the addition of tryptamine (data for this experiment have been deposited in the MASSIVE database, MSV000101919).

### Tryptamine derivatives produced as a general defense response

**Figure 6:**
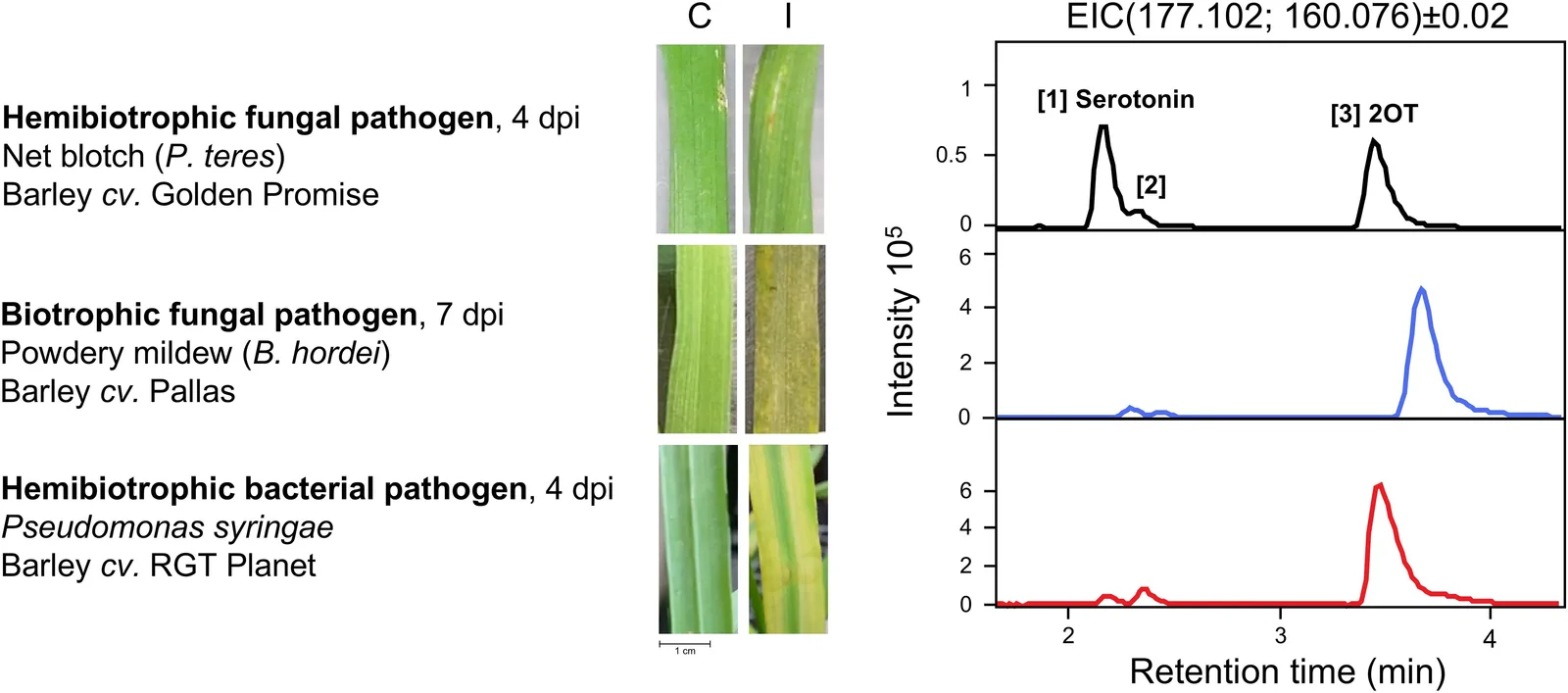
Induction of hydroxylated tryptamine derivatives in representative barley leaves of 3 different cultivars in response to infection by a biotrophic and 2 different hemibiotrophic plant pathogens. Methanolic extracts of barley leaves were analyzed by LC-MS, and representative extracted ion chromatograms (EICs) of ions *m/z* 177.102 and 160.076, corresponding to dominant ions of hydroxylated tryptamine derivatives, are shown. Induced production of [1] serotonin, [2] an unknown hydroxylated tryptamine derivative, and [3] 2OT was observed in barley leaf tissue from cv. Golden Promise infected with *P. teres* (black), cv. Pallas infected with powdery mildew (*B. hordei*) (blue), and cv. RGT Planet infected with *P. syringae* (red). Representative pictures of a control (c) and infected (i) leaf illustrate symptom development for each plant–pathogen interaction.

To assess whether serotonin and 2OT production represent a common defense response in barley, additional pathogens and cultivars were tested. Specifically, *P. teres* was also applied to cv. Golden Promise, cv. RGT Planet was infected with the hemibiotrophic bacterium *Pseudomonas syringae*, and cv. Pallas was infected with the biotrophic fungus *Blumeria hordei*. Disease symptoms characteristic of the respective pathogens were observed (Fig. 6), verifying effective infection. All studies show production of serotonin, 2OT, and a hydroxylated tryptamine derivative [2] (Fig. 6, Table S5). 2OT was consistently induced to relatively high levels across all treatments (Fig. 6, Table S5). Discrepancies in retention times between corresponding peaks are attributable to LC-MS analyses being performed on different days. In all LC-MS analyses, peak identities were confirmed using authentic standards. Interestingly, the typically susceptible cv. Golden Promise (Alhashel et al 2021) exhibited minimal disease-related symptoms after *P. teres* infection, and throughout disease development, the absolute levels of serotonin, hydroxylated tryptamine derivative [2], and 2OT were consistently lower than what was measured in the more affected cv. RGT Planet (Table S5). Ratios between the 3 hydroxylated tryptamine isomers varied across the experiments. Generally, more serotonin and the hydroxylated tryptamine derivative [2] were produced relative to 2OT levels during *P. teres* infection compared to leaves treated with *P. syringae* and *B. hordei*. Overall, these findings demonstrate that the induction of 2OT and related metabolites occurs broadly in barley–pathogen interactions, albeit with variation in their relative abundance.

### Endogenous metabolism of tryptamine, 2OT, and serotonin

The potential antimicrobial activity of 2OT and serotonin against *P. teres* was investigated with a spore inhibition assay where spores of *P. teres* were incubated with 1 or 5 mM concentrations of serotonin, 2OT, and hordenine, a known barley defense alkaloid with reported antimicrobial properties (Ishiai et al 2016), for 24 hours before being plated. After 5 d, fungal growth was evaluated, and in contrast to hordenine, neither 2OT nor serotonin showed any significant inhibitory effect under these conditions (Fig. S15). Therefore, it was hypothesized that 2OT and serotonin may be further metabolized. To investigate this hypothesis, barley leaves were supplied with exogenous tryptamine, 2OT, serotonin, or water. All fed substrates were still present after 24 h, but novel metabolites were observed for each substrate compared to the water control. Metabolite fold change was calculated against the control, and compounds with a fold change above 2 and a *P* value below 0.05 were identified and cross-referenced against the molecular network of infected barley leaves (Fig. 2).

When fed with exogenous tryptamine, 2 features corresponding to hydroxylated tryptamine isomer [2] and 2OT were observed (Table 1). Notably, no increase in serotonin levels was detected. Two additional features, *m/z* 193.10 and *m/z* 705.19, showed fold increases of 123 and 3, respectively. The feature with *m/z* 193.10 matched the theoretical mass of dihydroxytryptamine but did not align with the retention time of an authentic standard for AEHI, a phytoalexin reported by Ishihara et al (2017). The unknown feature with *m/z* 705.19 was not present in the molecular network.

**Table 1:** Metabolite features produced in barley leaves supplied with tryptamine, 2OT, and serotonin. If a feature was also identified in the barley metabolomics network (Fig. 2), the feature ID from the molecular network is noted. The fed substrates are not included.

Exogenous compound	*m/z*	RT^a^ (min)	ANOVA *P* value	Fold change (max/min)	Manual annotation	Feature iD	Notes
Tryptamine	160.0772	2.32	1.20E-02	33	Hydroxylated tryptamine derivative [2]	1,551	
	177.1032	2.32	1.90E-02	55		1,550	
	177.1029	3.41	4.20E-02	5	2OT	1,763	
	193.0979	3.6	3.90E-02	123	Unknown	1,791	*m/z* matches
							Dihydroxytryptamine
	705.1865	1.14	2.00E-03	3	Unknown	N/A	
2OT	146.0608	1.59	3.00E-02	19	AEHI	1,279	(Ishihara et al. 2017)
	193.0974	1.59	3.10E-02	50		1,276	
Serotonin	452.2035	2.13	6.70E-05	670	Unknown	1,486	
	191.0822	2.15	1.20E-03	27	Unknown	1,493	*m/z* matches *α/β-oxo-serotonin*
	221.093	2.14	1.30E-03	28	Unknown	1,485	

^a^RT, retention time.

In leaves infiltrated with 2OT, only a feature corresponding to the *m/z* and retention time of AEHI was observed, suggesting that barley can metabolize 2OT into AEHI.

In extracts from leaves infiltrated with serotonin, 3 features at *m/z* 452.20, *m/z* 191.08, and *m/z* 221.09 showed increased levels (Table 1). While the structures of these features were not elucidated, their presence in the molecular network supports the hypothesis that barley produces these compounds in response to *P. teres* infection.

## Discussion

### Barley metabolomic response to *P. teres* infection

The application of untargeted metabolomics is a pivotal tool within plant science research to enhance the understanding of metabolic reconfiguration in response to various stressors, thereby contributing to improved crop resilience and yield (Bozza et al 2024). A major challenge within untargeted LC-MS–based metabolomics relates to compound identification due to the vast number of unknown compounds (Giera et al 2022; Muñoz-Hoyos and Stam 2023). Here we used FBMN, which is a part of the GNPS2 platform, for global visualization and chemical annotation of our generated mass spectrometry dataset. In molecular networking, experimental mass spectra from detected chromatographic features are aligned against one another and connected based on spectral similarity. Each feature is depicted as a node in the network, and related nodes are grouped in MFs (Nothias et al 2020). FBMN can distinguish between isomers with very similar MS2 fragmentation spectra and was a useful tool to differentiate between 3 key hydroxylated tryptamine derivatives induced after pathogen infection (Fig. 2). Additionally, by combining the generated FBMN with in silico annotation tools (eg SIRIUS), the network nodes can function as an anchor to annotate structurally similar metabolites in the same MF and help decode the large unknown chemical space.

Here, 16.5% of metabolites were annotated, representing a relatively high annotation rate for an untargeted metabolomics dataset (De Jonge et al 2022). A large proportion of barley features (83.5%), however, remain unannotated. Given that many unknown metabolites are strongly induced in infected samples (eg MF 13 and MF 15; Fig. S16), much remains to be elucidated regarding the barley metabolic defense response. However, it should be noted that some of the unannotated metabolic space may not represent unknown metabolites but instead reflect in-source fragmentation (Giera et al 2024). Additionally, biochemically, very similar metabolites sometimes produce fragmentation patterns that are so different that they do not cluster in the same MF in the network, thus adding another layer of complexity to the annotation process. In our study, these challenges were exemplified by the parent ion for the phytoalexin AEHI (*m/z* 193.09 [M + H]^+^) being present as a singleton, while the in-source fragment ion *m/z* 146.06 for AEHI actually is connected to the identified hydroxylated tryptamine isomers in MF 3 (Fig. 2a, b; Table S1). Moreover, the in-source fragment ion was initially wrongly annotated as isoquinolone. The correct annotation as AEHI was therefore only possible because of the availability of an authentic standard.

The metabolomic pipeline employed in this study shows that barley produces a wide arsenal of specialized metabolites, including alkaloids, terpenoids, hydroxynitrile glucosides, and phenylpropanoids (Fig. 2). Some metabolites, such as the hydroxynitrile glucosides, hordatines, and hordenine, were unaffected by the biotic stress applied here, while other annotated metabolite classes were induced. Specifically, indole alkaloids and, in particular, tryptophan-derived metabolites, hydroxycinnamic acid amides (eg *p*-CHDA, feruloyltyramine, sinapoylagmatine, and *N*-feruloylserotonin), and phenylpropanoids, were induced after fungal infection. The metabolites produced and the metabolic shift in response to infection are in line with other studies (von Röpenack et al 1998; Ishihara et al 2017; Lemcke et al 2021; Ube et al 2021; Hamany Djande et al 2023). For example, aromatic amino acid metabolism and phenylpropanoid and isoquinoline alkaloid biosynthesis are induced in the *P. teres*–susceptible barley cv. Hessekwa (Hamany Djande et al 2023). Recently, several barley-specific labdane-related diterpenoids, hordedanes, were identified as important phytoalexins in response to fungal infections (Liu et al 2024). These metabolites were observed by Liu and colleagues (2024) in root exudates but were not identified in our leaf methanol extracts, indicating that the production of hordedanes is root-specific.

### Tryptophan-derived metabolites play an important role in barley defense response

With regard to the hypothesized tryptophan-derived metabolites, tryptamine and 3 hydroxylated tryptamine isomers were identified to be strongly induced after infection in multiple barley cultivars and in response to multiple plant pathogens with different pathogenic lifestyles (Figs. 2c, 6). Two of the hydroxylated tryptamine derivatives were confirmed to be serotonin and 2OT (Fig. 3). The identity of the third hydroxylated tryptamine isomer remains unknown. Ishihara et al (2017) reported the induction of a di-hydroxylated tryptamine derivative at positions 2′ and 3′ (AEHI) in barley after pathogen infection, demonstrating that barley has the capacity to hydroxylate tryptamine at the 3′ carbon. This suggests that the unidentified hydroxylated tryptamine derivative could potentially be 3-hydroxytryptamine. Furthermore, the metabolic network identified many features with an indolic backbone, including 3-methyl-2-oxindole, indoline, and *N*-methyltryptamine (Table S1, Fig. 2). These data support the hypothesis that tryptophan is critical for executing defense during barley infection. While this study assesses the nonvolatile metabolome of barley, volatile organic compounds (VOCs) have also been shown to play an important role in how barley responds to pathogen infection. Nonanal and β-ionone are emitted from barley leaves infected with *P. syringae* pv. *japonica* and are proposed to trigger a plant-to-plant SAR defense response (Brambilla et al 2022). In line with this result, we putatively identified glucosylated conjugates of β-ionone and other related β-ionone-like terpenoids, which may be the storage form of β-ionone prior to emission. Based on the strong induction of the genes related to indolic compounds, it would be highly relevant to perform untargeted VOC analysis of barley and other cereals to assess whether indole-related metabolites are both emitted and stored. Indeed, volatile indole is an important VOC following herbivore attack in maize (Erb et al 2015) and rice (Zhuang et al 2012).

### FMO and CYP monooxygenases capable of tryptamine hydroxylation in barley

Through a combination of heterologous expression in tobacco and *E. coli*, 2OTS was shown to catalyze the conversion of 2OT. It is possible that 2OTS hydroxylates tryptamine at the 2′ position, which then tautomerizes to 2OT in a lactam–lactim interconversion, as reported for oxindole (Kaur et al 2016). The identified 2OTS is a class B FMO from the so-called N-OX superfamily (Christensen and Neilson 2025; Jiang et al 2025; Vijayanathan et al 2025), one of several major FMO families in plants. Recent cross-kingdom evolutionary analysis of class B FMOs further classifies the N-OX superfamily as belonging to a diverse FMO clade (clade IV) comprising almost exclusively plant species but also containing an FMO from the protozoan *Trypanosoma cruzi* (Nicoll and Mascotti 2023). Other plant FMO families include the YUCCAs (clade III) and S-OXs (clade II), as well as a single Baeyer-Villiger monooxygenase reported from the moss *Physcomitrella patens* (Beneventi et al 2013; Thodberg and Jakobsen Neilson 2020; Nicoll and Mascotti 2023). The N-OX family was historically named based on previously characterized FMOs shown to hydroxylate nitrogen in small molecules (Thodberg et al 2020). This naming was proposed on the function of only 2 FMOs: FOS1 from ferns that performs 2 subsequent *N*-hydroxylations on phenylalanine, leading to phenylacetaldoxime as part of cyanogenic glycoside biosynthesis (Thodberg et al 2020), and *Arabidopsis* FMO1 (*At*FMO1) that catalyzes *N*-hydroxylation of pipecolic acid to form N-OH pipecolic acid, eliciting a SAR signaling response (Chen et al 2018b; Hartmann et al 2018). Heterologous expression in *N. benthamiana* has also demonstrated substrate promiscuity, such that *At*FMO1 can hydroxylate a second small molecule putatively identified as *N-*hydroxylated proline (Holmes et al 2019). Since then, other N-OX FMOs have been characterized, shown to perform both *N*- and *C*-hydroxylations. An N-OX3 FMO was recently discovered in Darwin's orchid (*Angraecum sesquipedale*) and is responsible for dual *N-*hydroxylations of phenylalanine and leucine to form their respective aldoximes as pollinator-attracting volatile scent molecules (Jiang et al 2025). The BX N-OXs from *A. squarrosa*, *L. galeobdolon*, and *C. orientalis* perform a dual *C-*hydroxylation on the C2 and C3 positions of indole, as intermediates in benzoxazinoid biosynthesis (Florean et al 2023; Florean et al 2025). Finally, an FMO from *Polygonum tinctorium* is capable of oxidizing indole to form indigo when expressed in *E. coli* (Inoue et al 2021). These findings necessitate a reevaluation of the assumption that FMOs from the N-OX family primarily hydroxylate nitrogen atoms and instead suggest that this family can perform both *C*- and *N*-hydroxylations on small aromatic and ring-like metabolites.

The biosynthetic pathway of serotonin in plants has been elucidated in rice, tomato, and green foxtail, all by enzymatic characterization of a CYP450 with tryptamine-5-hydroxylase activity (Fujiwara et al 2010; Commisso et al 2022; Dangol et al 2022). A BLAST of *Hv*CYP71P10 identified in this study showed that it shared 87%, 80%, and 55% sequence identity with OsCYP71P1 (rice), *Sv*T5H (green foxtail), and *Sl*T5H (tomato), respectively. Cytochrome P450 nomenclature dictates that enzymes sharing 55% identity belong to the same subfamily, placing all the genes in the CYP71P subfamily (Nelson, 2006). In barley, the function of CYP71P10 has previously been indirectly characterized by the genetic analyses of the *nec3* mutant, a disease lesion mimic mutant with impaired serotonin biosynthesis (Ameen et al 2021). Confirmation of CYP71P10 enzymatic function via heterologous expression in yeast was unsuccessful, so the tryptamine-5-hydroxylase capability of CYP71P10 reported here opens avenues for further characterization studies using the *nec3* mutant.

### Potential roles of serotonin

Here we show serotonin is significantly induced at 4 dpi, with levels increasing as the *P. teres* infection progressed. Serotonin has previously been found to be produced in response to pathogens in barley and other grasses (Ishihara et al 2008, 2017; Powell et al 2017; Lemcke et al 2021). The defense-related roles attributed to serotonin in Poaceae are multifaceted. Our best understanding of the role of serotonin in barley is facilitated by the *nec3* mutant, which develops atypically large necrotic lesions when exposed to a variety of penetrative pathogens. It has therefore been postulated that serotonin is a negative regulator of programmed cell death via an interaction with reactive oxygen species and links to cuticle stability (Ameen et al 2021). In rice, serotonin accumulates at sites of hypersensitive response lesions and is therefore proposed to help confine oxidative damage through its antioxidant activity, thereby preventing excessive cell death beyond infection sites (Hayashi et al 2016).

A similar necrotic lesion phenotype is also observed in a rice mutant devoid of serotonin, known as the *sl* (Sekiguchi lesion) mutant that lacks a functional *Os*CYP71P1 (Fujiwara et al 2010). Rice *sl* mutants have enhanced pathogen susceptibility and cannot deposit so-called “brown material” (Ishihara et al 2008). The ability to produce the brown material is restored after supplementation with serotonin, and by supplementing infected *sl* mutants with radiolabeled serotonin, 80% was deposited into the cell wall. Taken together, these results suggest that serotonin could also function as a key metabolite in physical cell wall fortification, helping to block pathogen entry. MALDI-MSI results show serotonin to be mainly localized at the sites of infection, which supports this hypothesis. Interestingly, trace levels of serotonin were also detected in the epidermis of uninfected barley leaves and support the findings within cuticle stability (Ameen et al 2021). The role of serotonin within biotic interactions is complex, such that plants may be more resistant to insect pests when serotonin biosynthesis is suppressed (Lu et al 2018).

Serotonin may also be involved in recruiting beneficial microbes during pest attack or infection. A recent study has shown that serotonin is a keystone metabolite exuded from switch grass roots during nutrient deficiency to recruit beneficial microbes (Baker et al 2024). Given that the induced systemic resistance is activated during infection by a hemibiotrophic pathogen, serotonin may likely also be exuded from barley roots to facilitate microbial interactions in a similar way. Overall, serotonin is an important cross-kingdom metabolite, and further investigations using genetic models are highly relevant for uncovering its role in plant–microbe interactions and systemic defense signaling.

### The potential roles of 2OT

The role of 2OT is, in its nature, unknown as the metabolite has not previously been reported in plants. We investigated 2 different hypotheses regarding the role of 2OT. The hypotheses were (i) 2OT is a phytoalexin induced after infection as a chemical defense response, and (ii) 2OT is an intermediate in the biosynthetic pathway of another defense compound.

To test the direct chemical defense capabilities of 2OT as a phytoalexin, we performed a spore inhibition assay against *P. teres*. The pathogen was exposed to differing concentrations of 2OT, serotonin, and hordenine. While hordenine exhibits clear inhibition of *P. teres* germination, no such inhibition was observed for either 2OT or serotonin. The lack of antimicrobial activity of these hydroxylated tryptamine derivatives questions their role as direct phytoalexins. Given that 2OT is structurally similar to serotonin, and MALDI-MSI shows that these 2 compounds colocalize during infection, it is possible that 2OT may be incorporated into the fortification of cell walls and/or the cuticle.

To investigate whether barley is able to further metabolize 2OT, we supplied healthy barley plants with exogenous 2OT through infiltration and analyzed changes in the metabolic profile compared with control water-fed plants. We observed a single compound increasing as a result of 2OT supplementation: the previously identified phytoalexin AEHI in barley (Ishihara et al 2017). This finding suggests that 2OT may be an intermediate for AEHI biosynthesis, which is supported by the colocalization of AEHI at the site of infection (Fig. S8). It is possible that 2OT may be an intermediate for other indolic compounds, but in this setup, the downstream catalyzing enzymes may not be available (ie they are only expressed when activated by specific defense gene cascades). For example, the detected serotonin-conjugates in our metabolic analysis were not measured in the feeding study. Additional validation obtained through radiolabeled precursor-feeding studies is needed to fully verify the identity of the biosynthetic precursors and downstream metabolism of 2OT and serotonin.

## Conclusions

In this study, we investigated the metabolic defense response of barley following infection by the hemibiotrophic fungal pathogen *P. teres*, the causal agent of net blotch. This disease poses a significant threat to global barley production, with yield losses of up to 40% reported (Clare et al 2020). Based on previous pathogenic studies, we hypothesized that tryptophan-derived specialized metabolism would play a key role in the barley defense response. Our results support this hypothesis, revealing a strong activation of tryptophan metabolism after *P. teres* infection. In particular, we identified serotonin and 2 hydroxylated tryptamine isomers as key metabolites induced at the infection site. One of these, 2OT, represents a previously unreported plant metabolite. We further demonstrate that serotonin and 2OT are biosynthesized from tryptamine by CYP71P10 and a flavin monooxygenase (2OTS) from the N-OX3 subfamily, respectively. These findings highlight the importance of tryptophan-derived specialized metabolism in barley defense, potentially contributing to cell wall fortification and the production of bioactive phytoalexins. This work lays a foundation for further exploration of metabolic defense pathways and may inform strategies for enhancing disease resistance in cereal crops.

## Materials and methods

### Barley plant material and cultivation

Three 2-rowed spring barley (*Hordeum vulgare*) cultivars—cv. Golden Promise, cv. RGT Planet, and cv. Pallas line P-02 (Kølster et al 1986)—were used. Seeds were imbibed overnight at 4 °C and germinated at a 1-cm depth in 1 row in soil. Plants were grown in a growth chamber (16 h light/8 h dark, 200 µE m^−2^ s^−1^, 19/16 °C day/night, 50% to 90% relative humidity).

### Pathogen inoculum preparation

*P. teres* (isolate CP2189), the causal agent of net form net blotch (Tini et al 2022), was grown on grass agar plates according to Jørgensen et al (1996). Conidia were washed off with sterile deionized water, and spore concentration was adjusted to 3,000 to 5,000 spores/mL according to Pandey et al (2021). *P. syringae* pv. *tomato* DC3000 were grown at 28 °C in LB medium supplemented with rifampicin (25 µg/mL) until they reached the late log phase. The bacteria were harvested by centrifugation (2,500 × *g*, 10 min), washed once in 10 mM MgCl_2_, and resuspended in 10 mM MgCl_2_ and 0.01% (v/v) Silwet L-77 adjusted to an optical density (OD) at 600 nm = 0.2 to 0.3. *B. hordei* (syn. *Blumeria graminis* f. sp. *hordei;* M. Liu et al 2021) isolate C15 (*AVR_A1_*) was maintained on cv. Pallas (P-02).

### Pathogen inoculation and sampling

Three days prior to inoculation with *P. teres* and *B. hordei*, the second developed barley leaves (BBCH 12; Lancashire et al 1991) were fixed as described by Jørgensen et al (1996). Fixed leaves were spray-inoculated with *P. teres* spore suspension until run-off or with *B. hordei* from sporulating cv. Pallas line P-02 plants as previously described (Jørgensen et al 1996). *P. syringae* was infiltrated into the abaxial side of the second forming leaf (BBCH 12; Lancashire et al 1991) by syringe injection as described previously (Katagiri et al 2002). For the systemic response experiment with *P. syringae*, leaves were infiltrated at the leaf tip and samples collected from the base of the leaf, 5 cm from the site of infiltration. Plants were covered in plastic bags directly after infection to ensure 100% relative humidity. Samples were collected as described in Table S6, snap-frozen in liquid N_2_ to quench metabolic activity, and stored at −80 °C until metabolite or RNA extraction.

### Metabolite extraction and untargeted metabolomic analysis

Frozen leaf tissue was homogenized using a Mixer Mill MM 400 (Retsch) (30 s, 30 Hz). Subsequently, 200 µL cold extraction buffer (85% methanol, 0.5% formic acid, 250 µM caffeine, and 250 µM sambunigrin) was added. Samples were vortexed, boiled (5 min, 80 °C), cooled on ice (10 to 20 min), and centrifuged (20,000 × *g*, 5 min, 4 °C). The supernatant was collected and stored at −80 °C. Prior to analysis, the supernatant was diluted 5 times, filtered through a 0.22-µM Millipore GV filter by centrifugation (3,000 rpm, 5 min, room temperature), and transferred to glass vials with 0.1-mL inserts. Throughout the sample preparation, samples were randomized to minimize bias. Untargeted metabolomic analysis was performed on a Dionex Ultimate 3,000RS UHPLC (Thermo Fisher Scientific) system connected to a compact quadrupole time-of-flight mass spectrometer (Bruker Daltonics) using electrospray ionization in positive mode (see supplementary Methods for specific details). QC samples (pooled aliquots of all plant extracts) and a process blank were included in the analysis to assess system stability and provide information on possible contaminants related to sample preparation, respectively.

### LC-MS data processing

The acquired raw spectral data were calibrated using the Bruker Compass DataAnalysis (v. 4.3) software (Bruker Daltonik GmbH 2014) and subsequently converted to open-source format *.mzML* files using msConvert (Chambers et al. 2012; Adusumilli and Mallick 2017. Hereafter, precursor *m/z* values were corrected by applying a freely available script on the *.mzML* files (Breaud et al., 2023; accessed at https://github.com/elnurgar/mzxml-precursor-corrector on 25 March 2025). The converted files were further processed using MZmine4 (v. 4.0.3) (Pluskal et al. 2010  Schmid et al. 2023) for feature detection, chromatogram building, feature deconvolution, alignment, and gap-filling. Briefly, a signal intensity noise cutoff of 200 and 100 was applied to MS1 and MS2 data, respectively. For scans in the retention time window of 0.5 to 15.0 min, chromatograms were built using the ADAP chromatogram algorithm with an *m/z* tolerance of 0.005 *m/z* (20 ppm) and a minimum absolute height of 2,000. The final aligned feature list was subjected to both Spectral/Molecular and Ion Identity networking and exported using the GNPS-FBMN/IIMN and SIRIUS/CSI-FingerID modules (Wang et al. 2016; Nothias et al 2020; Schmid et al. 2021). MS-DIAL version 5.1 (Tsugawa et al 2015; Takeda et al 2024) was used for processing LC-MS data from methanol extracts of barley leaves exogenously treated with tryptamine, 2OT, serotonin, or water (control). A detailed description of all parameters used for metabolite annotation and metabolomics data processing in MS-DIAL, MZmine4, FBMN, and SIRIUS in this study can be found in the supplementary Methods and Table S7.

### MALDI-TOF-MS

Fresh infected and control barley leaves were harvested at 4 dpi and embedded using an aqueous mixture of hydroxypropyl-methylcellulose and polyvinylpirrolidone (Mw 360 kDa) [7.5% (w/v) + 2.5% (w/v), respectively] and cross-sectioned (14 µm) in a Leica CM3050S Cryostat at −25 °C using Kawamoto's film method (Kawamoto and Kawamoto 2021) adapted for plant samples (Montini et al 2020). Intact sections were mounted onto glass slides using double-sided FrozenSTUCK cryo-tape (Labtag), then freeze-dried under vacuum overnight and stored under vacuum. MALDI matrix 2,5-dihydroxybenzoic acid (DHB) was applied as a 7.5-mg/mL DHB solution in ethanol/water/formic acid (90:10:0.1) by spraying with the HTX TM-Sprayer (HTX Technologies). Sections with applied matrix were analyzed on the same day. Matrix-deposited sections were imaged using the timsToF-fleX MALDI-2 instrument (Bruker Daltonics) with postionization applied (MALDI-2). The imaging was performed with a 20-µm spatial resolution, with a scan range of *m/z* 50 to 1,000. The procedure used 30 shots/pixel and a “Single” laser geometry, with an active beam scan and a trigger delay of 10 µs for the MALDI-2 postionization. Images were generated using SciLS Lab version 2024a Pro software (Bruker Daltonics) with a ±0.005 Da error interval to the exact mass, with root mean square normalization used.

### Structural elucidation of 2OT

Leaf material from barley cv. RGT Planet inoculated with *P. teres* was sampled in bulk 7 dpi and snap-frozen in liquid N_2_. Frozen leaf tissue was homogenized and metabolites extracted using a hot extraction buffer [70% methanol, 65 °C; 10:1 (w:w)] for 2 min. The extract was filtered through a Büchner funnel coupled with filter paper (Ø 85 mm), centrifuged (5 min, 4,200 rpm, 7 °C), and concentrated using a rotary evaporator, then freeze-dried. The complete pellet was dissolved in 10% (v:v) ethanol to match the starting conditions during flash chromatography purification. 2OT was isolated using a PuriFlash 5.250 coupled with an ELSD detector using an Uptisphere Strategy PF-15C18HQ-F0025 Interchim column (100 Å, 15 μm C18, 224 × 36 mm; Interchim). A binary solvent system consisting of water (A) and ethanol (B) was used for the gradient elution at a flow rate of 15 mL per minute. The gradient elution profile was as follows: 0.0 to 15.0, 5% to 40% B; 15.0 to 17.0, 40% to 100% B; 17.0 to 25.0, 100% B; 25.0 to 28.0, 100% to 5% B; and 28.0 to 32.0, 5% B. Fractions were collected at 3 different wavelengths (280, 240, and 210 nm). Collected fractions were combined, concentrated under N_2_, and freeze-dried. Structure elucidation was performed by NMR spectroscopy (see supplementary Methods material for NMR conditions).

### Chemical synthesis of *N*-hydroxytryptamine and 3-(2-aminoethyl)-1h-indol-1-ol

N-hydroxytryptamine was chemically synthesized according to a published procedure (Hee et al. 1992). 3-(2-Aminoethyl)-1H-indol-1-ol was chemically synthesized from the commercially available tryptamine. Thus, the primary amino group was first protected as the phthalimido derivative using a described procedure (Chantana et al 2021), and then the NH of the indole moiety was hydroxylated using the m-chloroperbenzoic acid method (Feenstrar̆ et al 1990), followed by deprotection of the primary amino group using the methylamine method (Motawia et al 1989) to provide the target molecule (see supplementary methods).

### RNA extraction and transcriptomic analysis

Total RNA was extracted from 25 to 60 mg leaf tissue using the Spectrum Plant Total RNA kit (Sigma-Aldrich). Transcriptomes were prepared from 36 samples consisting of 4 biological replicates per treatment (infected and mock control) and time point (1, 2, 4, and 7 dpi), representing 9 distinct sample conditions, including the T0 no-treatment control. RNA quality and integrity were assessed by NanoDrop and Bioanalyzer 2100/GX. Library preparation and sequencing were performed by Biomarker Technologies (BMK) GmbH using an Illumina NovaSeq X to generate 30-M paired-end reads of 150 bp (9 Gbp per sample). The raw sequencing data were processed by Sequentia Biotech. The FastP tool (Chen et al 2018a) was used for read quality filtering and trimming, and Kraken (Wood and Salzberg 2014) was used to classify the trimmed reads and remove unwanted rRNA-specific sequences. High-quality reads were then aligned against the cv. RGT Planet genome (v2) (Jayakodi et al 2024) using STAR (Dobin et al 2013) with default parameters. Gene expression was quantified using FeatureCounts and normalized with fragments per kilobase million, trimmed mean of M values, and transcript per million normalization. Lowly expressed genes were removed in R using the HTSFilter package (Rau et al 2013). Differential gene expression analysis was performed on the remaining 20,065 filtered genes using DESeq2 (Love et al 2014).

### Transient expression and substrate feeding in *N. benthamiana*

All genes were commercially synthesized (Genscript) with an N-terminal 6xHis tag and flanking attL sites in pUC57 (Thermo Scientific), functioning as Gateway entry vectors. Expression vectors were generated by Gateway recombination with PEAQ-HT-DEST1 and gene-containing entry vectors. PEAQ-HT (empty vector) was used as a control (Sainsbury et al 2009). Expression clones were verified by whole-plasmid sequencing (Plasmidsaurus; Azenta & UnveilBio). Verified plasmids were transformed into electro-competent *A. tumefaciens* strain AGL-1 (Lazo et al 1991). Transformed *Agrobacteria* were grown in Yeast Extract Peptone (YEP) medium (25 µg/mL rifampicin, 50 µg/mL carbenicillin, 50 µg/mL kanamycin) overnight at 28 °C. Cells were harvested by centrifugation (4,000 × g, 10 min), resuspended in infiltration buffer (pH 5.6; 50 mM MES, 2 mM sodium phosphate, 0.01% Silvet L-77, 0.5% D-sucrose, 200 µM acetosyringone) to OD_600_ = 0.8, and incubated at RT for 1 to 3 h. *Agrobacterium* suspensions were injected using a 1-mL syringe onto the abaxial leaf surface of 4- to 5-week-old *N. benthamiana* plants and placed in a greenhouse (21 °C day/19 °C night) for 4 d before 100 µM tryptamine in infiltration buffer was injected. After 24 h, 3 leaf discs from separate leaves from 1 plant were pooled, flash-frozen in liquid N_2_, and stored at −80 °C until metabolite extraction.

### Heterologous expression and crude enzyme preparation of 2OTS

The 2OTS gene (codon optimized) was cloned into pNIC28-Bsa4 (Savitsky et al 2010) and transformed into NiCo21 pRARE2 (DE3) *E. coli*. A single colony was grown overnight (37 °C, 250 rpm) in 50 mL TB medium (50 µg/mL kanamycin, 25 µg/mL chloramphenicol). Expression cultures (2 L TB medium) were inoculated with 20 mL seed culture in 5-L baffled flasks and incubated (37 °C, 250 rpm) until OD_600_ = 1.2 to 1.5 before induction with 0.5 mM isopropylthio-β-galactoside (18 °C; 150 rpm; 16 to 18 h). Cells were centrifuged (4 °C, 5,000 × *g*, 15 min) and resuspended in lysis buffer [100 mM Tris pH 7.5, 300 mM NaCl, 1 mM MgCl_2_, 0.5 mM tris(2-carboxyethyl)phosphine (TCEP), 0.04 mg/mL lysozyme, protease inhibitor (1 tablet/50 mL buffer), 25 U/mL Benzonase, and 0.05% NP-40] (5 mL/g cells), incubated for 30 to 60 min, and sonicated on ice (output control: 5, amplitude: 40%, 5 s on, 25 s off, 10 min). The lysate was then centrifuged (10,000 × *g*, 45 min), and the supernatant was collected and used for the enzyme assay. Empty NiCo21 pRARE2 (DE3) cells were processed identically and utilized as controls to assess background enzyme activity. SDS-PAGE was used for qualitative analysis, and the quantity was assessed by NanoDrop.

### 2OTS in vitro enzymatic assay

Enzymatic activity of 2OTS was assessed using crude enzyme. The assay (50 µL) contained 250 µM tryptamine, 10 µM FAD, 500 µM NADPH, 25 µL Tris-HCl buffer (pH 7.5), 5 mg crude 2OTS enzyme, and water. Controls lacked cofactors or enzyme; empty-vector extracts were also tested. The assay was carried out at 30 °C (300 rpm), stopped by the addition of methanol to a final concentration of 60%, and centrifuged (10,000 × g; 1 min) to avoid any precipitation in the sample. The assays were carried out in triplicate.

### Substrate feeding in *H. vulgare*

The first developed leaf of 1.5-leaf-stage barley cv. RGT Planet plants was infiltrated (abaxial side) with 100 µM substrate solution (tryptamine, serotonin, or 2-oxo-tryptamine) using a needleless 1-mL syringe. Control plants were fed with demineralized water. Twenty-four hours after feeding, 3 biological replicates for each substrate were sampled, flash-frozen in liquid N_2_, and stored at −80 °C until metabolite extraction. Measured products were compared as fold change between the substrate-fed and water-fed control.

### Spore inhibition assay

The inhibitory effect on spores of serotonin, 2OT, and hordenine (1 and 5 mM) was tested against the fungal phytopathogen *P. teres*, using demineralized water (2% methanol, 0.2% DMSO) as a negative control, as previously described (Shetty et al 2007). Different compound and concentration combinations were tested in triplicate. The final reaction had a spore concentration of 3,000 spores/mL, 2% methanol, and 0.2% DMSO. The reaction mixture was vortexed and subsequently incubated (RT, 200 rpm) for 24 h. Hereafter, 200 µL was plated on V8 agar plates using sterile glass beads. After 5 days at RT, fungal growth was visually inspected.

## Supplementary Material

kiag622_Supplementary_Data

## Data Availability

The raw *.d* and converted *.mzML* UHPLC-qTOF-MS/MS data files were uploaded to the MassIVE repository under accession number MSV000097403 in designated folders “Raw” and “mzML,” respectively. This repository also contains (i) a *.txt* metadata file with information related to each sample on treatment, time point, and fresh weight sample mass [mg]; (ii) ReDU metadata (Jarmusch et al 2020) enabling co- and reanalysis with publicly available data in the GNPS environment; (iii) a *.csv* file containing combined annotation results from GNPS, SIRIUS, CANOPUS, and MZmine; and (iv) a document with information on MZmine4, FBMN, and SIRIUS data-processing parameters. The FBMNs generated in GNPS2 within this study can be accessed with and without analog search. UHPLC-qTOF-MS/MS data files from extracts from transient expression of candidate monooxygenases in *N. benthamiana* were deposited as raw .*d* files under the MassIVE repository with accession number MSV000101919. Raw RNA sequencing data have been deposited in the NCBI Sequence Read Archive (SRA) under BioProject accession number PRJNA1460584. The data were generated using paired-end sequencing.
