# Supplementary material for: Biosynthesis of pathogen-induced hydroxylated tryptamine derivatives in barley
Source: Plant Physiol. 2026 Aug 22;202(1):kiag622. doi: 10.1093/plphys/kiag622 (PMC13579334; doi:10.1093/plphys/kiag622)
Supplement: Supplementary file 1 [file kiag622_supplementary_data.zip › SupplementalFigures_29052026_PlantPhys.docx]

Supporting Information

List of figures

**Figure S1:** Data processing workflow used for metabolite profiling

**Figure S2:** Assessment of reliability and quality of UHPLC-qTOF-MS/MS data acquisition.

**Figure S3:** Relative abundance of detected features in the chemical space of infected and non-infected barley leaves.

**Figure S4:** Principal component analysis of metabolites extracted from *P. teres* infected, mock control, or non-treated barley leaves across experimental time course.

**Figure S5:** Chromatographic comparison between infected and control barley samples through experimental time course.

**Figure S6:** NMR HSQC NMR fingerprint comparison of 2OT authentic standard and purified 2OT from *P. teres* infected barley leaf sample.

**Figure S7:** MALDI2 tims analysis of serotonin and 2OT

**Figure S8:** Random Forest analysis of barley *cv.* RGT Planet infected with *P. teres* at 4 days post infection.

**Figure S9:** Localization of tryptamine and hydroxylated tryptamines (serotonin and 2-oxo-tryptamine (2-OT)) in infected and control samples of barley leaves.

**Figure S10:** Localization of phytoalexins identified in random forest analysis in infected and control samples of barley leaves.

**Figure S11:** Localization of phytoanticipins hordenine ([M+K]+, *m/z* 204.0785), dihydroosmaronin ([M+K]+, *m/z* 300.0844), osmaronin ([M+K]+, *m/z* 298.0687) and sutherlandin ([M+H]+, *m/z* 276.1077) in infected and control samples of barley leaves using MALDI-MSI.

**Figure S12:** Localization of N-hydroxy-pipecolic acid ([M-NH3+H]+, *m/z* 129.0546), N-hydroxy-pipecolic acid glucoside ([M-NH3+H]+, *m/z* 291.1074) and pipecolic acid glucoside ([M+Na]+, *m/z* 314.1210; ([M-NH3+H]+, *m/z* 275.1125)) in cross-sections of infected and control samples of barley leaves using MALDI-MSI with spatial resolution of 20µm.

**Figure S13:** Co-expression analysis of genes involved in biosynthesis of tryptophan and tryptophan-derived defense metabolites.

**Figure S14:** Novel features detected by LC–MS analysis in plants expressing candidate monooxygenase genes

**Figure S15:** Evaluation of *P. teres* fungal growth after five days following spore inhibition assay.

**Figure S16:** Visual representation of annotated chemical space in barley leaves after infection by *P. teres*.

**Figure S1: Data processing workflow** used for the metabolite profiling and annotation of the acquired spectral data set from barley leaf methanolic extracts.

**Figure S2: Assessment of reliability and quality of UHPLC-qTOF-MS/MS data acquisition.** A principal component analysis (PCA) of the acquired full spectral dataset showed narrow clustering of quality control (QC) pooled samples (brown) and process blanks (PB; grey).

**Figure S3: Relative abundance of detected features in the chemical space of infected and non-infected barley leaves.** Global feature-based molecular network (FBMN) visualizing the detected metabolic landscape of barley *cv.* RGT Planet. The 38 largest molecular families (i.e. ≥ 3 nodes) in the network are numbered. Pie-charts indicate relative abundance of individual features in control (blue) and *P. teres* infected (orange) leaf samples across entire spectral dataset.

**Figure S4: Principal component analysis of metabolites extracted from *P. teres* infected, mock control, or non-treated barley leaves across experimental time course.** The principal component (PCA) scores plot for PC1 and PC2 **(a)** show strong separation of infected (orange) from mock control (blue) and non-treated (green) samples from 4 days post inoculation (dpi) with *P. teres*, while the PCA scores plot for PC3 and PC4 **(b)** show a distribution of the different sample types driven by ontogenetic development. The PCA loadings plots **(c) and (d)** show the features driving the distribution of the samples.

**Figure S5: Chromatographic comparison between infected and control barley samples through experimental time course.** Base peak chromatograms of representative methanol extracts of barley leaves infected with *P. teres* (blue) and control (treated with water; gray) at 1, 2, 4 and 7 days post inoculation (dpi). Serotonin, 2OT and tryptamine are labelled and verified by authentic standards.

**Figure S6:** **HSQC NMR fingerprint comparison** **of 2OT authentic standard and purified 2OT from *P. teres* infected barley leaf sample.** This figure shows the HSQC NMR spectra of the 2OT authentic standard reference (blue and green) and 2OT purified from a *P. teres* infected barley leaf sample (net blotch; red and magenta) for **(a)** the aromatic, and **(b)** the aliphatic region of the compound. The correlations in the HSQC spectra confirms that the purified compound from the infected barley leaf sample matches the authentic standard.

**Figure S7. MALDI2 tims analysis of serotonin and 2OT. (A)** Tims analysis of serotonin and 2OT [M-NH3+H]+ ions show an overlapping mobility value **(B)**The unique 2OTS ion [M+H]+ with a mobility value of 0.65 corresponded to a weak signal intensity in the infected tissue, with differential localization between the two metabolites inconclusive. Scale bar: 1mm

**Figure S8: Localization of tryptamine and hydroxylated tryptamine derivatives (serotonin and 2-oxo-tryptamine (2-OT)) in infected and control samples of barley leaves.** Serotonin and 2OT are detected as isobaric signals corresponding to [M+H]^+^ (*m/z* 177.1025) and [M-NH_3_+H]^+^ (*m/z* 160.0757) ions. Tryptamine signals correspond to [M+H]^+^ (*m/z* 161.1073) and [M-NH_3_+H]^+^ (144.0808) ions. AEHI signals correspond to [M+H]+ (m/z 146.0603). All signals are predominantly detected in infected regions, indicated by red squares. Scanned images of infected and control samples cross-sections are shown in top row of the figure. Color bar shows intensity of detected ions with yellow and blue colors corresponding to higher and lower intensity, respectively. All images are generated with ± 5mDa error interval.

**Figure S9: Random Forest analysis of barley *cv.* RGT Planet infected with *P. teres* at 4 days post infection. (a)** Metric multi-dimensional scaling (MDS) plot of the proximity matrix obtained from Random Forest (RF) analysis showing significant differences in the metabolome of control (blue) and infected (orange) barley leaves at 4 dpi with an out-of-bag (OOB) prediction error of 0%. **(b)** Top 15 important variables explaining the differences in the metabolome obtained from RF analysis are shown, and information on these variables/features are provided in Table S2.

**Figure S10: Localization of phytoalexins identified in random forest analysis in infected and control samples of barley leaves.** Each compound is assigned with a unique ID representing the feature ID in the molecular network. The features investigated were identified among top 15 most important variables contributing to the variance between *P. teres* infected and control barley leaves at 4 dpi in a Random Forest (RF) analysis (Figure S6 and Table S2). All signals are predominantly detected in infected regions, indicated by red squares. Scanned images of infected and control samples cross-sections are shown in top row of the figure. Color bar shows intensity of detected ions with yellow and blue colours corresponding to higher and lower intensity, respectively. All images are generated with ± 5mDa error interval.

**Figure S11: Localization of phytoanticipins hordenine ([M+K]^+^, *m/z* 204.0785), dihydroosmaronin ([M+K]^+^, *m/z* 300.0844), osmaronin ([M+K]^+^, *m/z* 298.0687) and sutherlandin ([M+H]^+^, *m/z* 276.1077) in infected and control samples of barley leaves using MALDI-MSI.** Compounds are dominantly detected in epidermal reagions of the analyzed barley leaves cross-sections, with exemption of hordenine, dominantly located in the mesophyll of infected regions. Scanned images of infected and control samples cross-sections are shown in top row of the figure. Color bar shows intensity of detected ions with yellow and blue colours corresponding to higher and lower intensity, respectively. All images are generated with ± 5mDa error interval.

**Figure S12: Localization of *N*-hydroxy pipecolic acid ([M-NH_3_+H]^+^, *m/z* 129.0546), *N*-hydroxy pipecolic acid glucoside ([M-NH_3_+H]^+^, *m/z* 291.1074) and pipecolic acid glucoside ([M+Na]^+^, *m/z* 314.1210; ([M-NH_3_+H]^+^, *m/z* 275.1125)) in cross-sections of infected and control samples of barley leaves using MALDI-MSI with spatial resolution of 20µm.** In the case of images with black border: the lack of signal is most likely caused by the low amount of the compounds in the analyzed replicate; Detection outside of tissue is most likely caused by matrix artefact, considering the uniform distribution pattern. Scanned images of infected and control samples cross-sections are shown in top row of the figure. Color bar shows intensity of detected ions with yellow and blue colours corresponding to higher and lower intensity, respectively. All images are generated with ± 5mDa error interval.

**Figure S13: Co-expression analysis of genes involved in biosynthesis of tryptophan and tryptophan-derived defense metabolites. (a)** Biochemical pathway for tryptamine, serotonin, and 2OT biosynthesis. ASα &Asβ (anthranilate synthase alpha/beta subunit), AnPRT (Anthranilate phosphoribosyltransferase), PRAI (Phosphoribosylanthranilate isomerase), IGPS (indole-3-glycerol-phosphate synthase), TSα & TSβ (Tryptophan synthase alpha/beta subunit), TDC (tryptophan decarboxylase), 2OTS (2-oxo-tryptamine synthase). **(b)** Tryptophan/tryptamine biosynthesis genes co-expression network generated in PlantNexus (Zhou et al., 2022). Mutual rank cutoff at ≤ 50. Red lines highlight the genes directly co-expressed with the FMO responsible for 2OT production (2OTS). **(c)** Log2(fold change) of tryptophan biosynthetic gene expression in barley plants infected with *P. teres* vs mock control at 1, 2, 4 and 7 dpi. GeneID is prefaced with ‘HORVU.RGT_PLANET.PROJ.’ for all genes.

**Figure S14:** Novel features detected by LC–MS analysis in plants expressing candidate monooxygenase genes compared to control plants expressing an empty pEAQ vector. Features were identified and visualized using MS‑DIAL. (Takeda et al., 2024)

 **Figure S15: Evaluation of P. teres fungal growth after five days following spore inhibition assay.** Spores of *P. teres* were incubated with 1 or 5 mM hordenine, 2OT, or serotonin for 24 hours, plated and incubated for five days at RT. Demineralized water (2% methanol, 0.2% DMSO) was used as control. 2OT and serotonin showed no inhibition compared to hordenine, which at 5 mM demonstrated clear inhibitory effect on fungal growth. Abbreviations: DW, demineralized water; DMSO, dimethyl sulfoxide; 2OT, 2-oxo-tryptamine; RT, room temperature.

**Figure S16: Visual representation of annotated chemical space in barley leaves after infection by *P. teres*. (a)** Global feature-based molecular network (FBMN) visualizing the detected chemical space of barley *cv.* RGT Planet. The 38 largest molecular families (i.e. ≥ 3 nodes) in the network are numbered. In this study, 16.5% of 922 features were annotated (highlighted in green). List of annotated compounds are found in Table S1. **(b)** Selected molecular families from network, which are induced in infected samples, but remain completely unannotated. At the top, pie-charts indicate relative abundance of individual features in control (blue) and infected (orange) samples across entire spectral dataset. Below, nodes are coloured according to compound class (NPC Pathway) predictions from SIRIUS. Abbreviations: NPC, NPClassifier taxonomy (Kim et al., 2021).

**References**

Kim, H. W., Wang, M., Leber, C. A., Nothias, L.-F., Reher, R., Kang, K. B., Van Der Hooft, J. J. J., Dorrestein, P. C., Gerwick, W. H., & Cottrell, G. W. (2021). NPClassifier: A Deep Neural Network-Based Structural Classification Tool for Natural Products. *Journal of Natural Products*, *84*(11), 2795–2807. https://doi.org/10.1021/acs.jnatprod.1c00399

Takeda, H., Matsuzawa, Y., Takeuchi, M., Takahashi, M., Nishida, K., Harayama, T., Todoroki, Y., Shimizu, K., Sakamoto, N., Oka, T., Maekawa, M., Chung, M. H., Kurizaki, Y., Kiuchi, S., Tokiyoshi, K., Buyantogtokh, B., Kurata, M., Kvasnička, A., Takeda, U., … Tsugawa, H. (2024). MS-DIAL 5 multimodal mass spectrometry data mining unveils lipidome complexities. *Nature Communications*, *15*(1), 9903. https://doi.org/10.1038/s41467-024-54137-w

Zhou, Y., Sukul, A., Mishler-Elmore, J. W., Faik, A., & Held, M. A. (2022). PlantNexus: A Gene Co-expression Network Database and Visualization Tool for Barley and Sorghum. *Plant and Cell Physiology*, *63*(4), 565–572. https://doi.org/10.1093/pcp/pcac007
