# Supplementary material for: Biosynthesis of pathogen-induced hydroxylated tryptamine derivatives in barley
Source: Plant Physiol. 2026 Aug 22;202(1):kiag622. doi: 10.1093/plphys/kiag622 (PMC13579334; doi:10.1093/plphys/kiag622)
Supplement: Supplementary file 1 [file kiag622_supplementary_data.zip › SupplementalMethods_29052026_PlantPhys.docx]

**Supplementary Methods:**

**Ultra-High Performance Liquid Chromatography**

Filtered methanol extracts were separated on a Dionex Ultimate 3000RS ultra-high performance liquid chromatography (UHPLC; Thermo Fisher Scientific, Waltham, Massachusetts, USA) system with a diode array detector. The autosampler and column were maintained at 10°C and 40°C, respectively. Four µl of extracts were injected onto and separated using a Phenomenex Kinetex® column (100 Å, 1.7 µm C18, 150 x 2.1 mm, Phenomenex Inc., Torrance, California, USA). Leucine-Enkephalin ([M+H]+ = 556.2771) was used as reference calibrant and injected in the beginning and end of the sample sequence to monitor sensitivity and performance of the UHPLC system. A binary solvent system consisting of water (A) and acetonitrile (B) both supplied with 0.05% (v/v) formic acid was used for the gradient elution at a flow rate of 0.3 mL per min. The gradient elution profile was as follows: 0.0-2.0 min, 2% B; 2.0-15.0 min, 2-40% B; 15.0-17.0, 40-100% B; 17.0-18.0, 100% B; 18.0-20.0, 100-2% B; 20.0-25.0, 2% B. The total run time for each sample was 25 min. In between each injection, the analytical column was allowed to re-equilibrate for 5 min.

The system stability was assessed using quality control (QC) samples consisting of pooled aliquots of all plant extracts. The QC samples were run in triplicate in the beginning and end of the total run as well as recurrently throughout acquisition. Injection order of extracts was randomized in order to minimize measurement bias. Samples were analyzed in batches of ten with each batch being separated by a system blank (100% methanol) and a QC sample. Additionally, a process blank (PB) sample was run in the beginning and end of the sample providing information on possible contaminants related to sample preparation. The PB sample was prepared following the same protocol as described but without any biological material.

**Mass spectrometry**

The UHPLC system was connected to a compactTM quadropole time-of-flight mass spectrometer (q-TOF MS) (Bruker Daltonics, Billerica, Massachusetts, USA). Electrospray ionization was used for ionization of the analytes in positive mode with an ion spray voltage maintained at 4000 V. End plate offset was set to 500 V. Nitrogen was used as the desolvation, nebulizing gas, and the collision gas. The desolvation gas temperature and flow rate was set to 250°C and 8 L/min, respectively. The nebulizing gas pressure was 2.0 bar. Mass (both MS1 and MS2) spectra were acquired in a scan range of 50 to 1400 m/z, scan time 0.3 s and collision energy at 7.0 eV. Active exclusion number was set to 3, and the precursor reconsideration time at 0.2 min; thus, after 3 MS/MS spectra had been recorded for a specific precursor ion it was excluded for analysis for the set time. The sampling rate was 4 Hz. Sodium formate (HCOONa) was used for internal mass calibration.

**Nuclear Magnetic Resonance (NMR)**

NMR samples were dissolved in DMSO-d6 or CDCl3 (calibrated to δH 2.50 ppm and δC 39.50 ppm or δH 7.26 ppm and δC 77.16 ppm, respectively) and 1D 1H, 13C and DEPT-135 spectra for synthetic compound references were acquired at 300 K on a Bruker Avance III spectrometer (1H operating frequency of 599.58 MHz) equipped with a Bruker SampleCase autosampler and a cryogenically cooled 13C/1H DCH 5-mm probe-head (Bruker Biospin) using standard pulse sequences and parameters (16 scans and a relaxation delay of 1 s for 1H and 256 scans and 2 s for 13C). 1D 1H, HSQC and HMBC spectra for minor metabolites (and 2OT reference) were acquired at 300 K on a Bruker Avance III spectrometer (1H operating frequency of 600.13 MHz) equipped with a Bruker SampleJet sample changer and a cryogenically cooled gradient inverse triple-resonance 1.7 mm TCI probe-head (Bruker Biospin) using standard 1D and 2D experiments (64 scans and 1730x256 data points for HSQC and 128 scans and 2048x256 data points for HMBC). Data was processed using Topspin ver. 4.4.0 (Bruker).

**MS-DIAL parameters**

Key parameters included MS1 and MS2 tolerances of 0.01 Da and 0.025 Da, respectively. Peak detection was performed with a minimum peak height of 3000 amplitude and a mass slice width of 0.1 Da. MS2Dec was configured with a sigma window value of 0.5 and an MS/MS abundance cut-off of 0. For feature alignment, retention time tolerance was set to 0.1 min and MS1 tolerance to 0.025 Da. Features showing a fold change (Max/Min) >2 and a p-value <0.05 relative to the control were manually inspected, annotated when possible, and cross-referenced with the FBMN derived from pathogen-infected barley leaves.

**GNPS parameters**

The GNPS export files (*.csv* quant table, *.mgf* MS/MS spectral summary, and *.csv* additional edges) generated in MZmine4 were uploaded to the GNPS2 platform. A feature-based molecular network (FBMN) was generated for the entire spectral dataset using the FBMN workflow (v. 2024.10.09) (Nothias, Petras et al. 2020). The precursor and fragment ion tolerance were set to 0.02 Da. For spectral similarity and spectral library search, the cosine score threshold was 0.7 with at least 6 matched fragment ions. When computing the FBMN, a task was submitted both with and without applying search for analogs to library spectra. The generated FBMN was visualized in Cytoscape (v. 3.10.2) (Shannon, Markiel et al. 2003).

**SIRIUS parameters**

The SIRIUS *.mgf* file containing spectral information for each feature generated in and exported from MZmine4 was processed with SIRIUS (v. 5.8.6) (Dührkop, Fleischauer et al. 2019). The instrument profile was set to q-TOF and the MS2 mass accuracy to 10 ppm. The prediction of the molecular formulas was improved by applying ZODIAC (Nothias et al., 2020), and structure predictions was done using CSI:FingerID and the COSMIC workflow (Dührkop et al., 2015; Hoffmann et al., 2022). CANOPUS was applied for *de novo* chemical compound class predictions using the NPClassifier taxonomy (Dührkop et al., 2015; Kim et al., 2021).

**Metabolite annotation**

In addition to publicly available spectral libraries (GNPS and MoNA), aAn in-house spectral library comprising nsisting of 11 reference compounds (Table S7; commercially sourced or in-house synthesized) analyzed with the same UHPLC-qTOF-MS/MS method as described above was used for metabolite identification. Reference compounds were analyzed with the same UHPLC-qTOF-MS/MS method as described above The reference compounds used within this study are listed in Table S7. The in-house spectral library and the library was generated usingin MZmine4 (v.4.0.3). using the Local compound database search and Batch spectral library generation modules. All compounds annotated in this study are listed in Table S1. Information on level of confidence for the identification of the individual metabolites is included. The confidence levels of identification referred to in this paper follows the guidelines by Metabolomics Standards Initiatives (Sumner et al., 2007) and the proposed modifications by Schymanski et al., 2014. The concentration of tryptophan, tryptamine, serotonin, hydroxylated tryptamine derivative [2], and 2OT in the plant extracts was quantified according to authentic standards.

**Metabolomic statistical analyses**

Features derived from blank samples were removed from the MZmine4 generated feature table using the Jupyter Notebook (R script) provided by (Pakkir Shah et al., 2023). Blank subtraction was done with a cutoff filter of 0.3, i.e. if the ratio of average intensity for any feature in blanks to samples were above 0.3, the feature was removed. Zeros present in the blank-removed feature table were randomly replaced by created random values between 0 and the minimum value in the blank-removed feature table. In R (v 4.4.3), feature intensities were normalized to mg fresh weight in the samples.

The supervised machine learning algorithm Random Forest (RF) (Breiman, 2001) was used to evaluate whether the metabolic space detected in our dataset could be separated by treatments (control versus infected) at 4 dpi. Furthermore, RF was used to obtain a list of variables most important for separation between treatments. The analysis was done in R (v 4.4.3) using the ‘randomForest’ function from the randomForest package (Liaw & Wiener, 2002) with ntree = 10.000 and mtry = 30.

The unsupervised learning method principal component analysis (PCA) was performed on the raw and on the blank-subtracted, imputed, and normalized spectral dataset. The analysis was done using the ‘pca’ function from the mdatools package with ncomp = 5, scale = TRUE, and center = TRUE. The results for RF and PCA were visualized using the R packages ggplot2 (Wickham, 2016) and mdatools (Kucheryavskiy, 2020).

**Supplementary Methods 2 -** Chemical synthesis of 3-(2-aminoethyl)-1H-indol-1-ol [1-Hyydroxy-1H-indole-3-ethanamine] (4):

**General procedures**

All reactions were carried out under an atmosphere of argon in oven-dried glassware with magnetic stirring. Unless otherwise indicated, commercially available starting materials, reagents, solvents and dry solvents were purchased from Sigma-Aldrich Chemicals and were used without further treatment. All commercial materials were used as received unless otherwise noted. All reactions were monitored by TLC on aluminum sheets coated with silica gel 60F254 (0.2 mm thickness, E. Merck, Darmstadt, Germany) using UV light as a visualizing agent. Column chromatographies were carried out using silica gel 60 (particle size 0.040-0.063 mm, 230-400 mesh ASTM, E. Merck). Solvent extracts were dried with anhyd. MgSO_4_ unless otherwise specified. Nuclear magnetic resonance (NMR) samples were dissolved in DMSO-*d*_6_ (calibrated to δ_H_ 2.50 ppm and δ_C_ 39.50 ppm )and 1D ^1^H, ^13^C and DEPT-135 spectra were acquired at 300 K on a Bruker Avance III spectrometer (^1^H operating frequency of 599.58 MHz) equipped with a Bruker SampleCase autosampler and a cryogenically cooled ^13^C/^1^H DCH 5-mm probe-head (Bruker Biospin, Karlsruhe, Germany) using standard pulse sequences and parameters (16 scans and a relaxation delay of 1 s for ^1^H and 256 scans and 2 s for ^13^C). Data were processed using Topspin ver. 4.4.0 (Bruker); coupling constants (*J*) are given in Hz. The multiplicities are reported as follows: singlet (s), doublet (d), doublet of doublets (td).

**Chemical synthesis of 3-(2-aminoethyl)-1H-indol-1-ol [1-Hyydroxy-1H-indole-3-ethanamine] (4).**

3-(2-Aminoethyl)-1H-indol-1-ol was chemically synthesized from the commercially available tryptamine, thus the primary amino group was first protected as the phthalimido-derivative using a described procedure (Chantana et al., 2021), then NH of the indole moiety was hydroxylated using m-chloroperbenzoic acid method (FeenstraR̆ et al., 1990) followed by deprotection of the primary amino group using methylamine-method (Motawia et al., 1989) to provide the target molecule as shown in the following Scheme:

**Synthesis of 2-(2-(1H-Indol-3-yl)ethyl)isoindoline-1,3-dione (3)[t the amino-protected tryptamine].**

The phthalimido-protected tryptamine **3** was synthesized in a good yield (98 %) according to a published procedure (Chantana et al., 2021).

A mixture of tryptamine derivatives **1** (1.60 g, 10.0 mmol), phthalic anhydride **2** (1.78 g, 12.0 mmol), and triethylamine (1.67 ml,12.0 mmol) in toluene (100 mL) was refluxed for 8 h. Following the reported procedure, desired product **3** (2.85 g, 98% yield) as a yellow solid (mp 168−170 °C); ^1^ H-NMR (600 MHz, DMSO-*d6*): δ 10.80 (s, 1H, indol NH), 7.83 (m, 4H, pthalimide-H), 7.54 (d, 1H, *J* = 7.6 Hz, indol-H), 7.32 (d, 1H; *J* = 7.6 Hz, indol-H), 7.16 ( d,1H, *J* = 2.3 Hz, indol-H), 7.05 (td, 1H, *J* = 7.6, 1.0 Hz, indol-H), 6.96 (td, 1H, *J* = 7.6, 1.0 Hz, indol-H), 3.85 (t, 2H, *J* = 7.6 Hz, -CH_2_*CH_2_*-Nphth), 3.02 (t, 2H, *J* = 7.6 Hz, , -*CH_2_*CH_2_-Nphth); ^13^C-NMR (125 MHz, DMSO-*d6*): : δ 167.9, 136.3, 134.4, 131.7, 127.1, 123.0, 123.0, 121.1, 118.4, 118.0, 111.5, 110.7, 38.3, 23.9. Those spectral data agreed with those previously reported in the

literature (Chantana et al., 2021)

**Chemical synthesis of 3-(2-aminoethyl)-1H-indol-1-ol [1-Hyydroxy-1H-indole-3-ethanamine] (4).**

A solution of 0.50 g (2.9 mmol) of 85% m-CPBA in CH_2_Cl_2_ (10.0 ml) is added dropwise to a cooled solution(ice/water) of **3** (0.63 g, 2.2 mmol) in CH_2_Cl_2_ (50.0 ml). After 3 hours the solvent evaporated and the residue is suspended in 99.9 % EtOH (50.0 ml). Then, a 33% solution of MeNH_2_ in absolute EtOH (25.0 ml) is added to the suspension of the residue at room temperature. After stirring for 5 min, the clear solution obtained is refluxed for 2.5 h. The mixture is cooled to room temperature, and the solvent is evaporated under reduced pressure. The residue is chromatographed on silica gel (40 g, 0.04-0.063 mm) using 5 % MeOH in CH_2_Cl_2_/MeOH (v/v) as eluent to remove all the impurities and then 1% HCOOH in MeOH (v/v) to obtain the desired product **4** as a pale yellow solid (0.092 g, 24 % yield); ^1^ H-NMR (600 MHz, DMSO-*d6*): δ 11.01 (s, 1H, 1-OH-1H-indol, exchangable), 8.41 (br s, 2H, exchangable), 7.54 (d, 1H, *J* = 7.6 Hz, indol-H), 7.36 (d, 1H; *J* = 7.6 Hz, indol-H), 7.21 ( br d,1H, *J* = 2.3 Hz, indol-H), 7.08 (td, 1H, *J* = 7.6, 1.0 Hz, indol-H), 6.99 (td, 1H, *J* = 7.6, 1.0 Hz, indol-H), 3.04 (br t, 2H, -CH_2_*CH_2_*-NH_2_), 2.99 (br t, 2H , -*CH_2_*CH_2_-NH_2_); ^13^C-NMR (125 MHz, DMSO-*d6*): δ 136.4, 126.9, 123.4, 121.2, 118.5, 118.2, 111.6, 109.8, 39.4, 23.4

r

**References**

Chantana, C., Sirion, U., Iawsipo, P., & Jaratjaroonphong, J. (2021). Short Total Synthesis of (±)-Gelliusine E and 2,3′-Bis(indolyl)ethylamines *via* PTSA-Catalyzed Transindolylation. *The Journal of Organic Chemistry*, *86*(19), 13360–13370. https://doi.org/10.1021/acs.joc.1c01461

Dührkop, K., Shen, H., Meusel, M., Rousu, J., & Böcker, S. (2015). Searching molecular structure databases with tandem mass spectra using CSI: FingerID. *Proceedings of the National Academy of Sciences*, *112*(41), 12580–12585.

FeenstraR̆, R. W., Stokkingreef, E. H. M., Reichwein, A. M., Lousberg, W. B. H., Ottenheijm, H. C. J., Kamphuis, J., Boesten, W. H. J., Schoemaker, H. E., & Meijer, E. M. (1990). Oxidative preparation of optically active n-hydroxy-α-amino acid amides. *Tetrahedron*, *46*(5), 1745–1756. https://doi.org/10.1016/S0040-4020(01)81979-7

Hoffmann, M. A., Nothias, L.-F., Ludwig, M., Fleischauer, M., Gentry, E. C., Witting, M., Dorrestein, P. C., Dührkop, K., & Böcker, S. (2022). High-confidence structural annotation of metabolites absent from spectral libraries. *Nature Biotechnology*, *40*(3), 411–421.

Jayakodi, M., Lu, Q., Pidon, H., Rabanus-Wallace, M. T., Bayer, M., Lux, T., Guo, Y., Jaegle, B., Badea, A., Bekele, W., Brar, G. S., Braune, K., Bunk, B., Chalmers, K. J., Chapman, B., Jørgensen, M. E., Feng, J.-W., Feser, M., Fiebig, A., … Stein, N. (2024). Structural variation in the pangenome of wild and domesticated barley. *Nature*, *636*(8043), 654–662. https://doi.org/10.1038/s41586-024-08187-1

Kim, H. W., Wang, M., Leber, C. A., Nothias, L.-F., Reher, R., Kang, K. B., Van Der Hooft, J. J. J., Dorrestein, P. C., Gerwick, W. H., & Cottrell, G. W. (2021). NPClassifier: A Deep Neural Network-Based Structural Classification Tool for Natural Products. *Journal of Natural Products*, *84*(11), 2795–2807. https://doi.org/10.1021/acs.jnatprod.1c00399

Motawia, M. S., Wengel, J., Abdel-Megid, A. E.-S., & Pedersen, E. B. (1989). A Convenient Route to 3′-Amino-3′-deoxythymidine. *Synthesis*, *1989*(05), 384–387. https://doi.org/10.1055/s-1989-27259

Nothias, L.-F., Petras, D., Schmid, R., Dührkop, K., Rainer, J., Sarvepalli, A., Protsyuk, I., Ernst, M., Tsugawa, H., Fleischauer, M., Aicheler, F., Aksenov, A. A., Alka, O., Allard, P.-M., Barsch, A., Cachet, X., Caraballo-Rodriguez, A. M., Da Silva, R. R., Dang, T., … Dorrestein, P. C. (2020). Feature-based molecular networking in the GNPS analysis environment. *Nature Methods*, *17*(9), 905–908. https://doi.org/10.1038/s41592-020-0933-6

Takeda, H., Matsuzawa, Y., Takeuchi, M., Takahashi, M., Nishida, K., Harayama, T., Todoroki, Y., Shimizu, K., Sakamoto, N., Oka, T., Maekawa, M., Chung, M. H., Kurizaki, Y., Kiuchi, S., Tokiyoshi, K., Buyantogtokh, B., Kurata, M., Kvasnička, A., Takeda, U., … Tsugawa, H. (2024). MS-DIAL 5 multimodal mass spectrometry data mining unveils lipidome complexities. *Nature Communications*, *15*(1), 9903. https://doi.org/10.1038/s41467-024-54137-w

Zhou, Y., Sukul, A., Mishler-Elmore, J. W., Faik, A., & Held, M. A. (2022). PlantNexus: A Gene Co-expression Network Database and Visualization Tool for Barley and Sorghum. *Plant and Cell Physiology*, *63*(4), 565–572. https://doi.org/10.1093/pcp/pcac007
